# Necklace: combining reference and assembled transcriptomes for more comprehensive RNA-Seq analysis

**DOI:** 10.1093/gigascience/giy045

**Published:** 2018-05-02

**Authors:** Nadia M Davidson, Alicia Oshlack

**Affiliations:** 1 Murdoch Childrens Research Institute, Royal Children's Hospital, Flemington Road, Parkville, Victoria 3052, Australia; 2 School of Bio-Sciences, University of Melbourne, Parkville, Victoria 3010, Australia

**Keywords:** transcriptome, assembly, RNA-seq, non-model

## Abstract

**Background:**

RNA sequencing (RNA-seq) analyses can benefit from performing a genome-guided and *de novo* assembly, in particular for species where the reference genome or the annotation is incomplete. However, tools for integrating an assembled transcriptome with reference annotation are lacking.

**Findings:**

Necklace is a software pipeline that runs genome-guided and *de novo* assembly and combines the resulting transcriptomes with reference genome annotations. Necklace constructs a compact but comprehensive superTranscriptome out of the assembled and reference data. Reads are subsequently aligned and counted in preparation for differential expression testing.

**Conclusions:**

Necklace allows a comprehensive transcriptome to be built from a combination of assembled and annotated transcripts, which results in a more comprehensive transcriptome for the majority of organisms. In addition RNA-seq data are mapped back to this newly created superTranscript reference to enable differential expression testing with standard methods.

## Findings

### Introduction

Despite the increasing number of species with a sequenced genome, the vast majority of reference genomes are incomplete. They may contain gaps, have unplaced assembly scaffolds, and be poorly annotated. The naïve approach to analyzing RNA sequencing (RNA-seq) on species with a genome would follow the same procedure as for model organisms, i.e., align reads the genome and count reads overlapping annotated genes, then test for differential expression based on gene counts [1]. However, this approach has the potential to miss important biology for many organisms. Segments of genes may be missed, either because of a gap in the reference sequence or missing annotation. The downstream differential expression analysis is likely to have reduced statistical power because the gene counts are underestimated. Similarly, we have observed different segments of a gene being annotated as separate genes such as when the gene spans multiple assembly scaffolds. However, this can happen even when a gene sits within a single scaffold. In the worst case, whole genes can be missed.

Ideally, an RNA-seq analysis could repair the gene models available from a reference genome and annotation by extracting information about the expressed genes from the data itself through genome-guided and/or *de novo* assembly [2]. However, analyses involving assembly remain complex, more so when multiple assemblies need to be integrated. Prior works such as that by Orgeur et al. [3] have gone some way to addressing the challenge; however, no reusable software has been written to perform these types of analyses.

In Davidson et al. 2017 [4], we introduced the concept of the superTranscriptome, where each gene is represented by one sequence containing all of that gene's exons in transcriptional order. SuperTranscripts provided a convenient means in which transcriptomes from difference sources, such as assembly and annotation, can be combined into a compact and unified reference. When applied to chicken, we showed that we could recover hundreds of segments of genes that were absent from the chicken reference genome.

Here, we present software, called Necklace, that automates the process described in [4] for any species with an incomplete reference genome. Necklace takes as input a configuration file containing paths to the RNA-seq reads, a reference genome, and one or more reference genome annotations. Because *de novo* assembly is error prone, we require that any gene discovered specifically through *de novo* assembly be also found among the coding sequence of a related (well-annotated) species. Therefore, the genome and annotation of a related species must also be provided to Necklace. Necklace will then run the steps involved in genome-guided and *de nov*o assembly and combine the assembled transcriptome with reference annotations for the species of interest. After building the superTranscriptome, Necklace will align and count reads in preparation for testing for differential gene expression and differential transcript usage using well-established tools such as edgeR [5], DEseq [6], or DEXseq [7].

In order to demonstrate the application of Necklace in a new dataset, we analyzed public RNA-seq data from sheep milk. Compared to using the sheep reference genome alone, the Necklace analysis resulted in 18% more reads being assigned to genes and 19% more differentially expressed genes being detected.

### The Necklace pipeline

**Figure 1:**
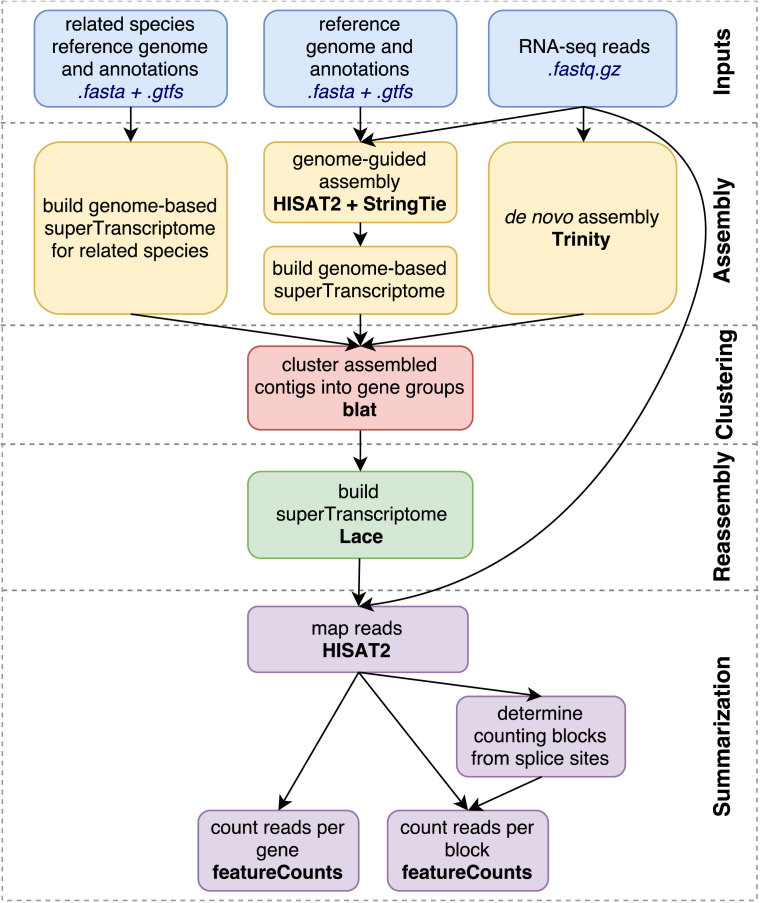
An overview of the Necklace pipeline. External software that Necklace runs is shown in bold.

Necklace is a pipeline constructed using the bpipe framework [8]. It steers external software, such as aligners and assemblers, as well as a set of its own utilities, written in C/C++. As input, Necklace takes the raw RNA-seq reads and the reference genome for the species as well as any available annotation. In addition, it takes a reference genome and annotation from a related but well-studied species such as human, drosophila, and yeast. Necklace consists of several sequential stages: initial genome-guided and *de novo* assembly, clustering transcripts into gene groupings, reassembly to build the superTranscriptome, and alignment and counting of mapped reads in preparation for differential expression testing and differential isoform usage testing. Each sequential stage consists of several substages and is outlined in Fig. 1 with further detail below.

### Assembly

The assembly stage creates three different transcriptomes. First reads are aligned to the reference genome using HISAT2 [9], and genome-guided assembly is performed with StringTie [10]. This assembly is combined with reference annotations and then flattened based on genomic location so that each exon is reported only once and overlapping exons are merged. Exonic sequence is then extracted from the genome and concatenated to build a “genome-based” superTranscriptome. We build the initial genome-based superTranscriptome rather than use a set of transcript sequences for two reasons. First, it results in the correct genomic order of a gene's exons. This ensures that when Lace is used in the reassembly step to combine the *de novo* assembled transcripts, the final superTranscriptome also has exons in the correct genomic order. Second, this step reduces the amount of sequence to be processed in the clustering and reassembly steps.

In parallel to building the genome-based superTranscriptome for the species of interest, the related species annotation is used to create a genome-based superTranscriptome (without genome-guided assembly). Finally, RNA-seq reads are *de novo* assembled with Trinity [11].

### Clustering of transcripts

This step assigns *de novo* assembled transcripts to gene clusters prior to building the final superTranscriptome. Those contigs that align to the genome-based superTranscriptome (using Blat [12]) are allocated to known genes, and those not aligning to the genome but found in the related species superTranscriptome are assigned to novel genes. *De novo* assembled contigs that are not found in either the genome-based superTranscriptome or a related species are filtered out. *De novo* assembled transcripts that align to more than one gene are also removed to avoid false chimeras [13] from being introduced into the superTranscriptome.

A limitation of this filtering is that *de novo* assembled contigs cannot be used to scaffold highly fragmented references because those contigs will give the appearance of false chimeras. Novel genes that are absent from the genome and the related species will also be missed with our approach. However, *de novo* assemblies are highly error prone, and strict filtering of assembled transcripts is required to eliminate the introduction of artifacts into the annotation.

### Reassembly of superTranscripts

Each cluster consists of a gene's genome-based superTranscript and/or its set of *de novo* assembled transcripts. The transcripts in each cluster are merged together through Lace assembly [4] to produce one superTranscript per gene.

### Summarization

Reads are aligned back to the superTranscriptome using HISAT2, and fragments are counted per gene using featureCounts [14]. Splice junctions reported by HISAT2 are used to segment each superTranscript into a set of contiguous “blocks.” Fragments are then counted in “blocks” and can be used for differential isoform detection such as exon counts.

### Application to differential expression testing in sheep transcriptomes

To demonstrate the utility of Necklace, we applied it to public RNA-seq from Churra sheep milk and compared transcriptome expression at day 10 to day 150 post lambing [15]. Necklace was given the sheep reference genome Oar_v3.1. This version of the sheep genome is 2.6 Gb in size, with 85 Mb of unfilled assembly gaps. It consists of 5,698 scaffolds (28 chromosomes and 5,670 unplaced contigs). Human, with the hg38 reference genome, was used as the related species. For both genomes, version 90 of the Ensembl annotation was used (see Methods section).

The Ensembl reference consisted of 29, 118 transcripts, and reference-guided assembly using StringTie resulted in 65, 717 transcripts. The sheep data was *de novo* assembled into 267, 553 contigs; however, only 63, 592 contigs were reassembled into the Necklace superTranscriptome due to filtering at the clustering step. The magnitude of this reduction is consistent with alternative clustering methods (e.g., the removal of contigs with little read support using Corset [13]).

Use of these data and set of reference files resulted in a more comprehensive transcriptome. Compared to the Ensembl sheep annotation, the number of bases included in the Necklace transcriptome increased by 76%,and 18% more reads were assigned to genes by featureCounts (Table 1). This more comprehensive reference included 2,208 (8%) more genes. A small subset of the novel genes (404 genes) was assembled in the antisense direction or overlapping an annotated Ensembl gene. Of the remaining novel genes, 1,718 (95%) had homology to sequence in the Ref-seq RNA database [16] with an expect value (E) below 10^−50^ when aligned using blastn [17]. The novel genes predominately matched coding (86%) rather than noncoding (14%) sequence. For 381 novel genes, an open reading frame of 100 or more amino acids was identified with homology to protein sequence from another species.

**Table 1:** A comparison of analysis using the Ensembl reference annotation alone and using the superTranscriptome generated by Necklace for our example sheep dataset

	Reference	Necklace
**Bases (Mbp)**	45.13	79.56
**Reads assigned to genes**	194, 693 ,051	230, 140, 801
**Genes**	26, 613	28, 821
**Differentially expressed genes**	383	456

**Figure 2:**
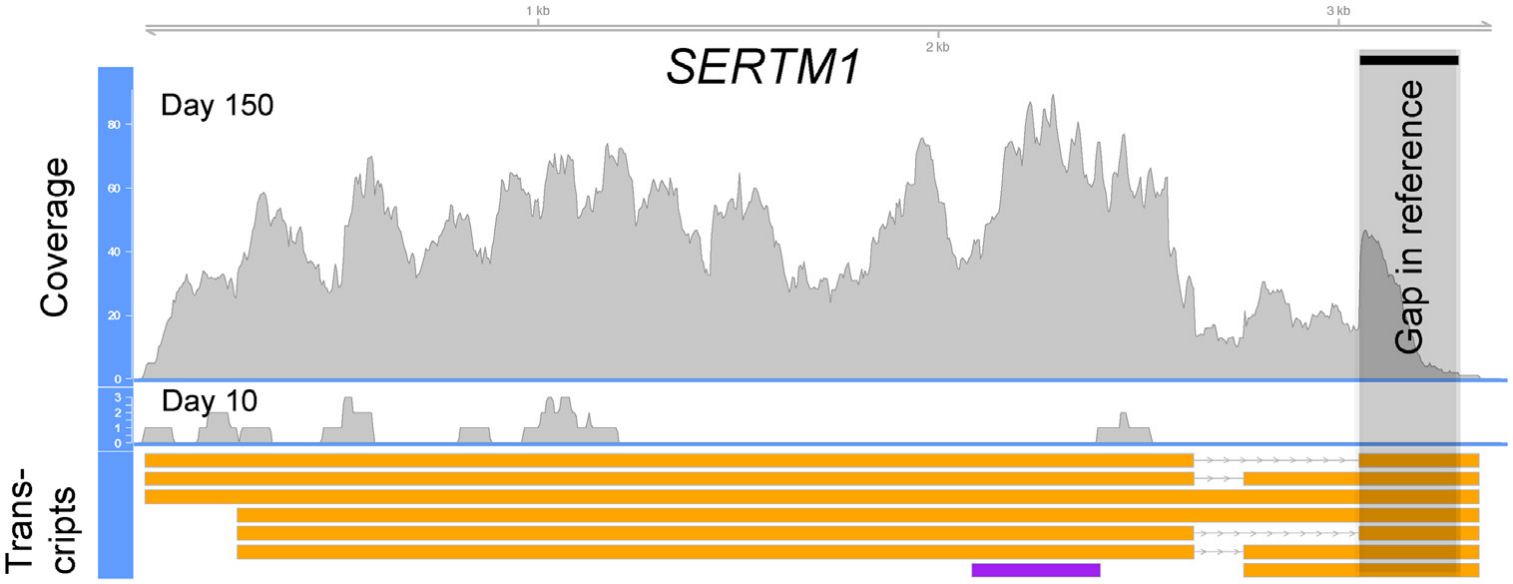
Read coverage aggregated over replicate samples for the Necklace assembled superTranscript of SERTM1. This gene is found to be significantly differentially expressed using the fragments per kilobase of transcript per million mapped reads Necklace generated reference but is missed when the reference genome and annotation are used in isolation due to low read counts. The reference annotation consists of a single transcript of 321 bp (shown in purple), whereas the *de novo* assembled gene consists of seven transcripts up to 3,333 bp long (shown in orange) and includes approximately 250 bp that are absent from the reference genome, in a location consistent with a genome assembly gap. The genome-guided transcripts that were assembled for this gene were filtered out with StringTie's merge function due to an average fragments per kilobase of transcript per million mapped reads <1.

When performing differential expression analysis using edgeR, the Necklace transcriptome identified more significantly differentially expressed genes than when the reference was used alone (456 compared to 383, false discovery rate [FDR] <0.05). Some of these differences could be attributed to the inclusion of novel unannotated genes, with 66 of the newly annotated genes identified as differentially expressed. A predicted protein coding gene could be assigned to 45 of the novel, differentially expressed genes after blastn alignment to the Ref-seq RNA database. Necklace was also able to improve the detection of differential expression among several known genes by providing more complete gene sequences. Larger numbers of reads mapping to the longer sequences resulted in more power for differential expression testing. One example of this was the *SERTM1* gene where the annotated transcript only included 321 bp, while the Necklace superTranscript contained 3,333 bp and overlapped a genome assembly gap (Fig. 2).

## Conclusion

Here, we present Necklace, a pipeline designed to improve RNA-seq analysis in species with an incomplete genome and annotation. We believe Necklace is the first pipeline to automate the steps required to combine reference and assembled data, alignment, and summarization of counts, thereby making the analysis process user friendly and reproducible.

Incorporating *de novo* assembled data into the analysis of species with a semicomplete genome promises to give a more comprehensive picture of the transcriptome. However, de novo assemblies can also introduce artifacts; for this reason, Necklace only adds *de novo* assembled transcripts that correspond to novel protein-coding genes that are found in a related species. Assembled transcripts that match multiple known genes are also removed in order to prevent the introduction of false chimeric sequences. Therefore, it is still possible that some *bona fide* expressed transcripts are not included in the final results.

In real data, we show that indeed applying Necklace to a sheep dataset resulted in a more complete transcriptome. We were able to discover more exon and more genes, some of which had homology to known protein-coding sequences in other species. This analysis also resulted in more power for differential expression analysis. Necklace is open source and available from github at [18] and has resource RRID:SCR_016103.

## Methods

### Data

Sheep RNA-seq data was downloaded from the Sequence Read Archive (accession numbers SRR2932539- SRR2932542, SRR2932561-SRR2932564). The sheep genome and annotation were downloaded from Ensembl [19, 20]. The human reference genome and annotation were downloaded from Ensembl [21, 22]. We then selected coding sequence from the human annotation using the command:

*grep " CDS " data/Homo_sapiens.GRCh38.90.gtf > Homo_sapiens.GRCh38.90.CDS.gtf*.

### Necklace analysis

For the Necklace analysis of sheep milk, all data files were placed into a subdirectory called “data.” A Necklace input file, “data.txt”, was created with the following lines:

  *// sequencing data*
  *reads_R1=“data/SRR2932539_1.fastq.gz*,
data/SRR2932540_1.fastq.gz,   data/SRR2932541_1.fastq.gz,
data/SRR2932542_1.fastq.gz,   data/SRR2932561_1.fastq.gz,
data/SRR2932562_1.fastq.gz,   data/SRR2932563_1.fastq.gz,
data/SRR2932564_1.fastq.gz”
  *reads_R2=“data/SRR2932539_2.fastq.gz,*
data/SRR2932540_2.fastq.gz,   data/SRR2932541_2.fastq.gz,
data/SRR2932542_2.fastq.gz,   data/SRR2932561_2.fastq.gz,
data/SRR2932562_2.fastq.gz,   data/SRR2932563_2.fastq.gz,
data/SRR2932564_2.fastq.gz”

  *//The genome and annotation*
  *annotation=“data/Ovis_aries.Oar_v3.1.90.gtf”*
  *genome=“data/Ovis_aries.Oar_v3.1.dna.toplevel.fa”*

  *//The genome and annotation of a related species*
  *annotation_related_species=“data/Homo_sapiens.GRCh38.90.CDS.gtf”*
  *genome_related_species=“data/Homo_sapiens.GRCh38.dna.toplevel.fa”*

Necklace version 0.9 was then run using the command:

*<necklace   path>/tools/bin/bpipe run -n 8 <necklace path>/necklace.groovy data/data.txt*

Version numbers of all the external tools that Necklace calls can be found in Necklace's installation script, “install_linux64.sh”.

Necklace was run on 8 cores on a 48 core AMD Opteron(tm) Processor 6180 SE 2.5 GHz CentOS 6.7 server with 252GB of RAM. The full run time was approximately 4.5 days.

The assembly stage took approximately 3.5 days due to the *de novo* assembly (Trinity [11] was run with six threads and 50 GB memory maximum). Genome-guided assembly was run concurrently on one core and took 20 hours. The time for clustering was 11 hours, which was dominated by alignment of the *de novo* assembled contigs to the related species with blat. Lace ran 13 hours, and read realignment and summarization took 1.5 hours.

### Annotation of novel genes

Novel superTranscript sequences were aligned to the Ref-seq RNA database (downloaded from [23]) using blastn version 2.2.25+ and the command:

*blastn –db refseq_rna –outfmt 6 –query novel_ST.fasta -num_alignments 1*

StringTie and Trinity sequence from novel sheep genes were extracted and analyzed with TransDecode [24] and blastp (BLASTP, RRID:SCR_001010) [25] against the UniProt (UniProt, RRID:SCR_002380) [26] database using the commands:

*TransDecoder.LongOrfs -t <transcripts.fasta>*

*blastp -query transdecoder_dir/longest_orfs.pep\*

*-db uniprot_sprot.fasta -max_target_seqs 1\*

*-outfmt 6 -evalue 1e-5 -num_threads 10 > blastp.outfmt6*

*hmmscan –cpu 8 –domtblout pfam.domtblout/path/to/Pfam-A.hmm transdecoder_dir/longest_orfs.pep*

*TransDecoder.Predict -t target_transcripts.fasta –retain_pfam_hits pfam.domtblout –retain_blastp_hits blastp.outfmt6*

### Reference-based analysis

To make the reference-based analysis as similar as possible to the Necklace pipeline, we used the versions of HISAT2 (HiSat2, RRID:SCR_015530), samtools (SAMTOOLS, RRID:SCR_002105), and featureCounts (featureCounts, RRID:SCR_012919) that were installed by Necklace.

HISAT2 was run on each sample using the command:

*hisat2 –known-splicesite-infile <splice sites file> -x <genome index> -1 <input_1.fastq.gz> -2 <input_2.fastq.gz> | samtools view -u—> <output.bam>*

Where the splice sites file and genome index were the same we used allignements from the initial stage of Necklace wherereads were aligned to the reference genome.

Reads were then counted for each annotated gene using featureCounts with the command:

featureCounts -T 8 –primary -p -t exon -g gene_id -a Ovis_aries.Oar_v3.1.90.flat.gtf -o counts *.bam

Where “Ovis_aries.Oar_v3.1.90.flat.gtf“ was a flattened version of the sheep Ensembl annotation and was created with the Necklace command:

   *gtf2flatgtf                        Ovis_aries.Oar_v3.1.90.gtf*

*Ovis_aries.Oar_v3.1.90.flat.gtf*

Flattening the annotation involves merging transcripts of a gene into a nonredundant but complete set of exons.

### Differential expression testing

For differential gene expression testing, gene-level counts were analyzed using the R bioconductor package edgeR (version 3.18.1) (edgeR, RRID:SCR_012802) [27]. We modeled both the time-point post lambing and animal in the design matrix:

*time_point←c(rep(“Day10”,4), rep(“Day150”,4))*

*indv←c(3141,4860,49 537,9539,3141,4860,9539,49 537)//numbers are animal IDs*

*design ← model.matrix(∼0+factor(indv)+factor(time_point))*

*colnames(design) ← gsub(“factor”, "", colnames(design))*

*sample_names=paste(indv, time_point, sep = “_”)*

*rownames(design) = sample_names*

The counts table was read into R and passed to edgeR:

*counts = count_table[,7: ncol(count_table)]*

*y ← DGEList(counts = counts)*

Genes with a counts per million (cpm) less than or equal to 0.5 in fewer than four samples were filtered out and the libraries normalized.

*keep ← rowSums(cpm(y) > 0.5) > = 4*

*y ← y[keep,, keep.lib.sizes = TRUE]*

*y ← calcNormFactors(y)*

We then estimated the dispersion and looked for differential expression with an FDR <0.05:

*y ← estimateDisp(y, design, robust = TRUE)*

*fit ← glmFit(y, design, robust = TRUE)*

*qlf ← glmLRT(fit, coef = 5)*

*is.de ← decideTests(qlf, p.value = 0.05)*

## Availability of supporting source code and requirements

Project name: Necklace

Scicrunch RRID:SCR_016103

Project home page: https://github.com/Oshlack/necklace/wiki

Operating systems: Linux

Programming language: Groovy and C/C++

Other requirements: Java 1.8

License: GPL 3.0

## Availability of supporting data

An archival snapshot of the code is available in the *GigaScience* GigaDB repository [28].

## Abbreviations

bp: base pair; cpm: counts per million; FDR: false discovery rate; RNA-seq: RNA sequencing.

## Supplementary Material

GIGA-D-17-00354.pdf

GIGA-D-17-00354_R1.pdf

GIGA-D-17-00354_R2.pdf

Response_to_Reviewer_Comments_Original_Submission.pdf

Response_to_Reviewer_Comments_Revision_1.pdf

Reviewer_1_Report_(Original_Submission) -- Li Song 1/12/2018 Reviewed

Reviewer_1_Report_(Revision_1) -- Li Song 3/25/2018 Reviewed

Reviewer_2_Report_(Original_Submission) -- Mickael Orgeur 1/16/2018 Reviewed

Reviewer_2_Report_(Revision_1) -- Mickael Orgeur 3/21/2018 Reviewed
