# Supplementary material for: Necklace: combining reference and assembled transcriptomes for more comprehensive RNA-Seq analysis
Source: Gigascience. 2018 May 2;7(5):giy045. doi: 10.1093/gigascience/giy045 (PMC5946861; doi:10.1093/gigascience/giy045)
Supplement: GIGA-D-17-00354.pdf [file giy045_giga-d-17-00354.pdf]

GigaScience

Necklace: combining reference and assembled transcriptomes for more comprehensive RNA-Seq analysis

--Manuscript Draft--

**Manuscript Number:**	GIGA-D-17-00354
**Full Title:**	Necklace: combining reference and assembled transcriptomes for more comprehensive RNA-Seq analysis
**Article Type:**	Technical Note
**Funding Information:**	National Health and Medical Research Council (GNT1126157) — Prof. Alicia Oshlack
**Abstract:**	Background: RNA-Seq analyses can benefit from performing a genome-guided and de novo assembly, in particular for species where the reference genome or the annotation is incomplete. However, tools for integrating assembled transcriptome with reference annotation are lacking.<br>Findings: Necklace is a software pipeline that runs genome-guided and de novo assembly and combines the resulting transcriptomes with reference genome annotations. Necklace constructs a compact but comprehensive superTranscriptome out of the assembled and reference data. Reads are subsequently aligned and counted in preparation for differential expression testing.<br>Conclusions: Necklace allows a comprehensive transcriptome to be built from a combination of assembled and annotated transcripts which results in a more comprehensive transcriptome for the majority of organisms. In addition RNA-seq data is mapped back to this newly created superTranscript reference to enable differential expression testing with standard methods. Necklace is available from https://github.com/Oshlack/necklace/wiki under GPL 3.0.
**Corresponding Author:**	Alicia Oshlack<br><br>AUSTRALIA
**Corresponding Author Secondary Information:**	
**Corresponding Author's Institution:**	
**Corresponding Author's Secondary Institution:**	
**First Author:**	Nadia Davidson
**First Author Secondary Information:**	
**Order of Authors:**	Nadia Davidson<br>Alicia Oshlack
**Order of Authors Secondary Information:**	
**Opposed Reviewers:**	
**Additional Information:**	
**Question**	**Response**
Are you submitting this manuscript to a special series or article collection?	No
**Experimental design and statistics**<br><br>Full details of the experimental design and statistical methods used should be given in the Methods section, as detailed in our	Yes

Powered by Editorial Manager® and ProduXion Manager® from Aries Systems Corporation

Minimum Standards Reporting Checklist. Information essential to interpreting the data presented should be made available in the figure legends.<br><br>Have you included all the information requested in your manuscript?	
**Resources**<br><br>A description of all resources used, including antibodies, cell lines, animals and software tools, with enough information to allow them to be uniquely identified, should be included in the Methods section. Authors are strongly encouraged to cite Research Resource Identifiers (RRIDs) for antibodies, model organisms and tools, where possible.<br><br>Have you included the information requested as detailed in our Minimum Standards Reporting Checklist?	Yes
**Availability of data and materials**<br><br>All datasets and code on which the conclusions of the paper rely must be either included in your submission or deposited in publicly available repositories (where available and ethically appropriate), referencing such data using a unique identifier in the references and in the “Availability of Data and Materials” section of your manuscript.<br><br>Have you have met the above requirement as detailed in our Minimum Standards Reporting Checklist?	Yes

Manuscript Click here to download Manuscript Necklace Gigascience.docx

Necklace: combining reference and assembled transcriptomes for more comprehensive RNA-Seq analysis

Nadia M Davidson[1,2,*] and Alicia Oshlack[1,2,*]
[1]Murdoch Childrens Research Institute, Royal Children's Hospital, Victoria, Australia
[2]School of Bio-Sciences, University of Melbourne, Victoria, Australia
*To whom correspondence should be addressed.
Contact: nadia.davidson@mcri.edu.au or alicia.oshlack@mcri.edu.au

Abstract

Background: RNA-Seq analyses can benefit from performing a genome-guided and de novo assembly, in particular for species where the reference genome or the annotation is incomplete. However, tools for integrating assembled transcriptome with reference annotation are lacking.
Findings: Necklace is a software pipeline that runs genome-guided and de novo assembly and combines the resulting transcriptomes with reference genome annotations. Necklace constructs a compact but comprehensive superTranscriptome out of the assembled and reference data. Reads are subsequently aligned and counted in preparation for differential expression testing.
Conclusions: Necklace allows a comprehensive transcriptome to be built from a combination of assembled and annotated transcripts which results in a more comprehensive transcriptome for the majority of organisms. In addition RNA-seq data is mapped back to this newly created superTranscript reference to enable differential expression testing with standard methods. Necklace is available from https://github.com/Oshlack/necklace/wiki under GPL 3.0.

Keywords: transcriptome, assembly, RNA-Seq, non-model

Findings

Introduction

Despite the increasing number of species with a sequenced genome, the vast majority of reference genomes are incomplete. They may contain gaps, have unplaced assembly scaffolds and be poorly annotated. Therefore, analyzing RNA-Seq data using just the reference genome has the potential to miss important biology for many organisms. Ideally, an RNA-Seq analysis could utilise prior knowledge of the gene-models available from a reference genome and annotation, whilst also extracting information about the genes from the data itself through genome-guided and/or de novo assembly [1,2].

In [3] we introduced the concept of the superTranscriptome, where each gene is represented by one sequence containing all of that gene's exons in transcriptional order. SuperTranscripts provided a convenient means in which transcriptomes from difference sources, such as assembly and annotation, can be

combined into a compact and unified reference. When applied to chicken, we showed that we could recover hundreds of segments of genes that were absent from the chicken reference genome.

Here we present software called necklace which automates the process described in [3] for any species with an incomplete reference genome. Necklace takes as input a configuration file containing paths to the RNA-Seq reads, a reference genome and one or more reference genome annotation. Because de novo assembly is error prone, we require that any gene discovered specifically through de novo assembly be also found amongst the coding sequence of a related (well annotated) species. Therefore, the genome and annotation of a related species must also be provided to necklace. Necklace will then run the steps involved in genome-guided and de novo assembly, and combine the assembled transcriptome with reference annotations for the species of interest. After building the superTranscriptome, necklace will align and count reads in preparation for testing for differential gene expression and differential transcript usage using well established tools such as edgeR [4], DEseq[5] or DEXseq[6].

In order to demonstrate the application of necklace in a new data set we analysed public RNA-seq data from sheep milk. Compared to using the sheep reference genome on it own, the necklace analysis resulted in 18% more reads being assigned to genes and 19% more differentially expressed genes being detected.

The Necklace pipeline

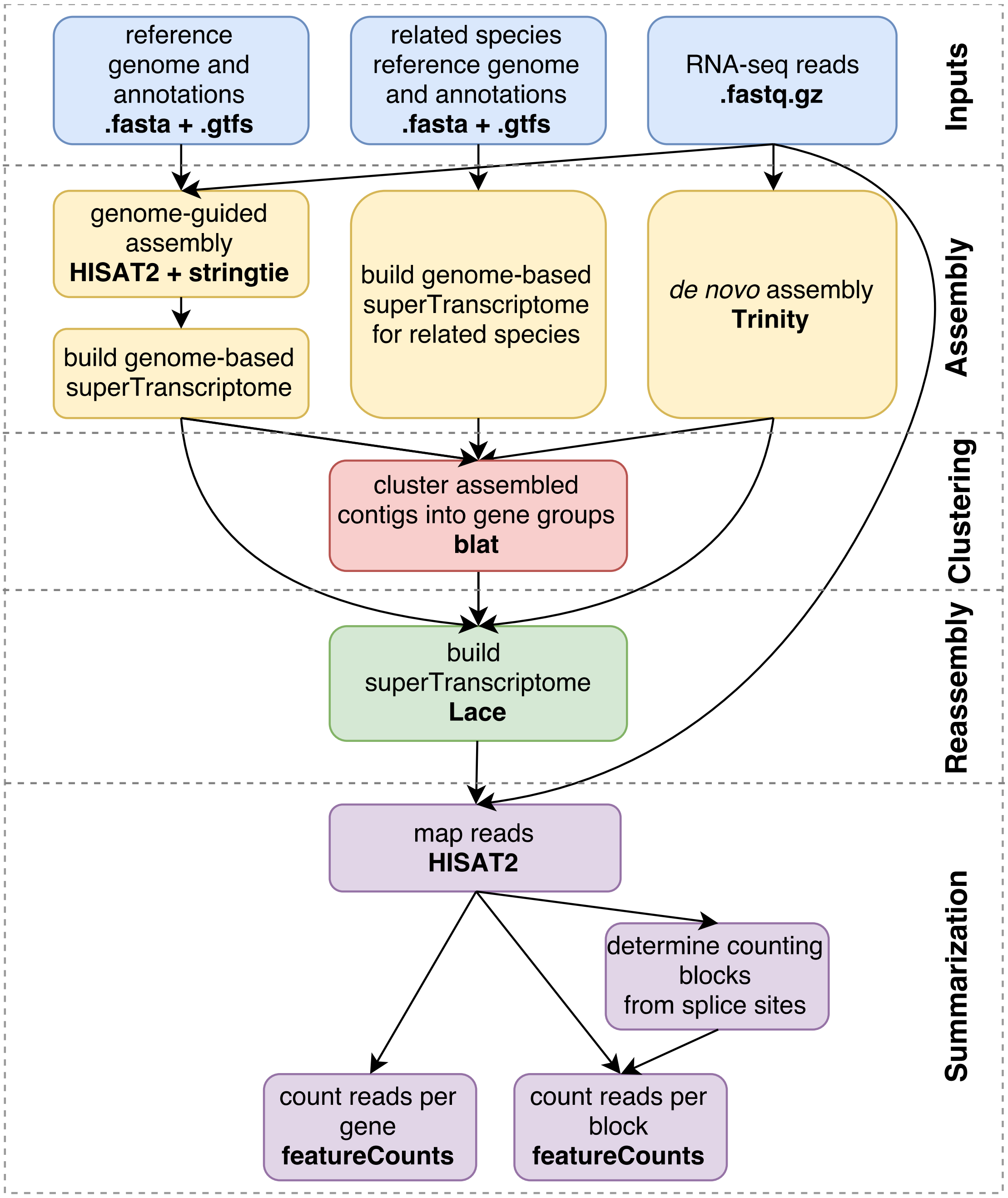

Figure 1. *An overview of the necklace pipeline. External software that necklace runs is shown in bold.*

Necklace is a pipeline constructed using the bpipe framework [7]. It steers external software, such as aligners and assemblers, as well as a set of its own utilities, written in c/c++. As input Necklace takes the raw RNA-seq reads and the reference genome for the species as well as any available annotation. In addition, it takes a reference genome and annotation from a related, but well studied species such as human, drosophila, yeast, etc. Necklace consists of several sequential stages: initial genome guided and *de novo* assembly, clustering transcripts into gene groupings, reassembly to build the superTranscriptome and finally alignment and counting of mapped reads in preparation for

differential expression testing and differential isoform usage testing. Each of these sequential stages consists of several sub-stages and is outlined in Figure 1 with further detail below.

1. **Assembly** – The assembly stage creates three different transcriptomes. First reads are aligned to the reference genome using HISAT2 [8] and genome-guided assembly is performed with StringTie [9]. This assembly is combined with reference annotations and then flattened based on genomic location, so that each exon is reported only once and overlapping exons are merged. Exonic sequence is then extracted from the genome and concatenated to build a "genome-based" superTranscriptome. In parallel, the related species annotation is used to create a "genome-based" superTranscriptome (without genome-guided assembly). Finally, RNA-Seq reads are de novo assembled with Trinity [10].
2. **Clustering of transcripts** – This step assigns de novo assembled transcripts to gene clusters prior to building the final superTranscriptome. Those contigs aligning to the genome-based superTrascriptome (using Blat [11]) are allocated to known genes while those not aligning to the genome, but found in the related species superTranscriptome are assigned to novel genes. De novo assembled transcripts that align to more than one gene are removed to avoid false chimeras [12] from being introduced into the superTranscriptome.
3. **Reassembly of superTranscripts** - Each cluster consists of a gene's genome-based superTranscript and/or its set of de novo assembled transcripts. The transcripts in each cluster are merged together through Lace assembly [3], to produce one superTranscript per gene.
4. **Summarization** - Reads are aligned back to the superTranscriptome using HISAT2 and fragments counted per gene using featureCounts [13]. Splice junctions reported by HISAT2 are used to segment each superTranscript into a set of contiguous "blocks". Fragments are then counted in "blocks" and can be used for differential isoform detection like exon counts.

Application to differential expression testing in sheep transcriptomes
To demonstrate the utility of necklace, we applied it to public RNA-Seq from Churra sheep milk and compared transcriptome expression at day 10 to day 150 post lambing [14]. Necklace was given the sheep reference genome, Oar_v3.1. Human, with the hg38 reference genome, was used as the related species. For both genomes, version 90 of the Ensembl annotation was used (see methods). Using this data and set of reference files resulted in a more comprehensive transcriptome. Compared to the Ensembl sheep annotation, the number of bases included in the necklace transcriptome increased by 76% and there were 2208 (8%) more genes identified. This more comprehensive reference resulted in 18% more reads being assigned to genes by featureCounts compared to the reference annotation alone. When performing differential expression analysis using edgeR the necklace transcriptome identified more significantly differentially expressed genes (456 compared to 383, FDR<0.05). Some of these differences could be attributed to the inclusion of novel unannotated genes with 66 of the newly

annotated genes being identified as differentially expressed. Necklace was also able to improve the detection of differential expression amongst several known genes by providing more complete gene sequences. Larger numbers of reads mapping to the longer sequences resulted in more power for differential expression testing. One example of this was the *SERTM1* gene where the annotated transcript only included 321bp while the necklace superTranscript contained 3333bp and overlapped a genome assembly gap (Figure 2).

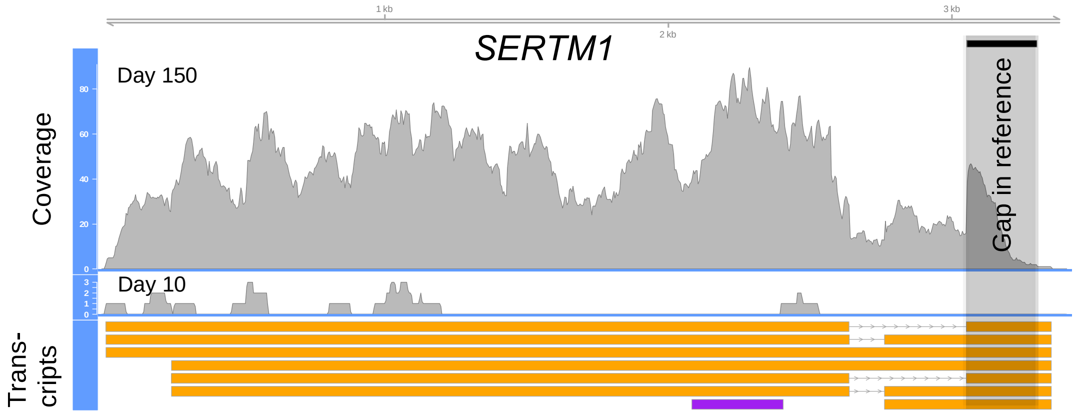

Figure 2. *Read coverage aggregated over replicate samples for the necklace assembled superTranscript of SERTM1. This gene is found to be significantly differentially expressed using the necklace generated reference, but is missed when the reference genome and annotation are used in isolation due to low read counts. The reference annotation consists of a single transcript of 321bp (shown in purple), whereas the de novo assembled gene consists of seven transcripts up to 3333bp long (shown in orange) and includes approximately 250bp that is absent from the reference genome, in a location consistent with a genome assembly gap. The genome-guided transcripts that were assembled for this gene were filtered out by stringTie's merge function due to an average FPKM < 1.*

Conclusion

Here we have presented necklace, a pipeline designed to improve RNA-Seq analysis in species with an incomplete genome and annotation. We believe necklace is the first pipeline to automate the steps required to combine reference and assembled data, alignment and summarization of counts. We show that this process results in more complete transcriptomes using a sheep data set and superTranscripts give more power for differential expression analysis.

Methods

Sheep RNA-Seq data was downloaded from SRA (accession numbers SRR2932539- SRR2932542,SRR2932561-SRR2932564). The sheep genome and annotation was downloaded from Ensembl:
ftp://ftp.ensembl.org/pub/release-90/fasta/ovis_aries/dna/Ovis_aries.Oar_v3.1.dna.toplevel.fa.gz
ftp://ftp.ensembl.org/pub/release-90/gtf/ovis_aries/Ovis_aries.Oar_v3.1.90.gtf.gz

The human reference genome and annotation was also downloaded from Ensembl:
ftp://ftp.ensembl.org/pub/release-90/fasta/homo_sapiens/dna/Homo_sapiens.GRCh38.dna.toplevel.fa.gz,
ftp://ftp.ensembl.org/pub/release-90/gtf/homo_sapiens/Homo_sapiens.GRCh38.90.gtf.gz

We then selected coding sequence from the human annotation using the command:

```
grep " CDS " data/Homo_sapiens.GRCh38.90.gtf > Homo_sapiens.GRCh38.90.CDS.gtf
```

For the necklace analysis of sheep milk, all data files were placed into a subdirectory called “data” and a necklace input file, “data.txt”, was created with the following lines:

```
// sequencing data
reads_R1="data/SRR2932539_1.fastq.gz,data/SRR2932540_1.fastq.gz,data/SRR2932541_1.fastq.gz,data/SRR2932542_1.fastq.gz,data/SRR2932561_1.fastq.gz,data/SRR2932562_1.fastq.gz,data/SRR2932563_1.fastq.gz,data/SRR2932564_1.fastq.gz"
reads_R2="data/SRR2932539_2.fastq.gz,data/SRR2932540_2.fastq.gz,data/SRR2932541_2.fastq.gz,data/SRR2932542_2.fastq.gz,data/SRR2932561_2.fastq.gz,data/SRR2932562_2.fastq.gz,data/SRR2932563_2.fastq.gz,data/SRR2932564_2.fastq.gz"

//The genome and annotation
annotation="data/Ovis_aries.Oar_v3.1.90.gtf"
genome="data/Ovis_aries.Oar_v3.1.dna.toplevel.fa"

//The genome and annotation of a related species
annotation_related_species="data/Homo_sapiens.GRCh38.90.CDS.gtf"
genome_related_species="data/Homo_sapiens.GRCh38.dna.toplevel.fa"
```

Necklace version 0.9 was then run using the command:

```
<necklace path>/tools/bin/bpipe run -n 8 <necklace path>/necklace.groovy data/data.txt
```

Version numbers of all the external tools that necklace calls can be found in necklace’s installation script, “install_linux64.sh”.

To make the reference based analysis as similar as possible to the necklace pipeline we used the versions of HISAT2, samtools and featureCounts that were installed by necklace.

HISAT2 was run on each sample using the command:

```
hisat2 --known-splicesite-infile <splice sites file> -x <genome index> -1 <input_1.fastq.gz> -2 <input_2.fastq.gz> | samtools view -u - > <output.bam>
```

Where the splice sites file and genome index were the same ones generated in the initial stage of necklace that aligns reads to the reference genome.

Reads were then counted for each annotated gene using featureCounts with the command:

```
featureCounts -T 8 --primary -p -t exon -g gene_id -a Ovis_aries.Oar_v3.1.90.flat.gtf -o counts *.bam
```

Where "Ovis_aries.Oar_v3.1.90.flat.gtf" was a flattened version of the sheep Ensembl annotation and was created with the necklace command:

```
gtf2flatgtf Ovis_aries.Oar_v3.1.90.gtf Ovis_aries.Oar_v3.1.90.flat.gtf
```

Flattening the annotation involves merging transcripts of a gene into a non-redundant but complete set of exons.

For differential gene expression testing, gene-level counts were analysed using the R bioconductor package edgeR (version 3.18.1) [15]. We modeled both the time-point post lambing and animal in the design matrix:

```
time_point<-c(rep("Day10",4),rep("Day150",4))
indv<-c(3141,4860,49537,9539,3141,4860,9539,49537) //numbers are animal IDs
design <- model.matrix(~0+factor(indv)+factor(time_point))
colnames(design) <- gsub("factor","",colnames(design))
sample_names=paste(indv,time_point,sep="_")
rownames(design)=sample_names
```

The counts table was read into R and passed to edgeR:

```
counts=count_table[,7:ncol(count_table)]
y <- DGEList(counts=counts)
```

Genes with a counts per million (cpm) less than or equal to 0.5 in less 4 samples were filtered out and the libraries normalized.

```
keep <- rowSums(cpm(y) > 0.5) >=4
y <- y[keep, , keep.lib.sizes=TRUE]
y <- calcNormFactors(y)
```

We then estimated the dispersion and looked for differential expression with a false discovery rate (FDR) < 0.05:

```
y <- estimateDisp(y,design,robust=TRUE)
fit <- glmFit(y, design,robust=TRUE)
qlf <- glmLRT(fit,coef=5)
is.de <- decideTests(qlf, p.value=0.05)
```

Availability of supporting source code and requirements

Project name: Necklace

Project home page: https://github.com/Oshlack/necklace/wiki

Operating system(s): Linux

Programming language: Groovy and C/C++

Other requirements: Java 1.8

License: GPL 3.0

Declarations

List of abbreviations

FPKM – fragements per kilobase of exon per million mapped reads

Competing interests

None declared.

Funding

AO is funded by an *NHMRC CDF GNT1126157.*

Authors' contributions

ND wrote all the software and drafted the paper. AO oversaw the project and contributed to writing the manuscript.

Acknowledgements

We would like to thank Anthony Hawkins, the author of Lace, who contributed to the early concept of necklace when applied to chicken.

References

1. Martin J, Wang Z. Next-generation transcriptome assembly. Nat. Rev. Genet. [Internet]. 2011;12:671–82. Available from: http://dx.doi.org/10.1038/nrg3068
2. Orgeur M, Martens M, Börno ST, Timmermann B, Duprez D, Stricker S. A dual transcript-discovery approach to improve the delimitation of gene features from RNA-seq data in the chicken model. Biol. Open [Internet]. The Company of Biologists Ltd; 2017 [cited 2017 Dec 18];bio.028498. Available from:

http://www.ncbi.nlm.nih.gov/pubmed/29183907
3. Davidson NM, Hawkins ADK, Oshlack A. SuperTranscripts: a data driven reference for analysis and visualisation of transcriptomes. Genome Biol. 2017 181 [Internet]. BioMed Central; 2017 [cited 2017 Sep 18];18:148. Available from: https://genomebiology.biomedcentral.com/articles/10.1186/s13059-017-1284-1
4. Robinson M, McCarthy D, Chen Y, Smyth GK. edgeR: differential expression analysis of digital gene expression data User→s Guide. 2011;
5. Anders S, Huber W. Differential expression analysis for sequence count data. Genome Biol. [Internet]. 2010 [cited 2013 May 21];11:R106. Available from: http://genomebiology.com/2010/11/10/R106
6. Anders S, Reyes A, Huber W. Detecting differential usage of exons from RNA-seq data. Genome Res. [Internet]. Cold Spring Harbor Laboratory Press; 2012 [cited 2016 Sep 14];22:2008–17. Available from: http://www.ncbi.nlm.nih.gov/pubmed/22722343
7. Sadedin SP, Pope B, Oshlack A. Bpipe: a tool for running and managing bioinformatics pipelines. Bioinformatics [Internet]. 2012 [cited 2013 Nov 15];28:1525–6. Available from: http://bioinformatics.oxfordjournals.org/content/early/2012/04/11/bioinformatics.bts167.abstract
8. Kim D, Langmead B, Salzberg SL. HISAT: a fast spliced aligner with low memory requirements. Nat. Methods [Internet]. 2015 [cited 2016 Sep 14];12:357–60. Available from: http://www.ncbi.nlm.nih.gov/pubmed/25751142
9. Pertea M, Pertea GM, Antonescu CM, Chang T-C, Mendell JT, Salzberg SL. StringTie enables improved reconstruction of a transcriptome from RNA-seq reads. Nat. Biotechnol. [Internet]. Nature Research; 2015 [cited 2017 Sep 18];33:290. Available from: http://www.nature.com/nbt/journal/v33/n3/full/nbt.3122.html
10. Haas BJ, Papanicolaou A, Yassour M, Grabherr M, Blood PD, Bowden J, et al. De novo transcript sequence reconstruction from RNA-seq using the Trinity platform for reference generation and analysis. Nat. Protoc. [Internet]. Nature Publishing Group, a division of Macmillan Publishers Limited. All Rights Reserved.; 2013 [cited 2013 Oct 30];8:1494–512. Available from: http://dx.doi.org/10.1038/nprot.2013.084
11. Kent WJ. BLAT--the BLAST-like alignment tool. Genome Res. [Internet]. 2002 [cited 2013 May 29];12:656–64. Available from: http://www.pubmedcentral.nih.gov/articlerender.fcgi?artid=187518&tool=pmcentrez&rendertype=abstract
12. Davidson NM, Oshlack A. Corset: enabling differential gene expression analysis for de novo assembled transcriptomes. Genome Biol. [Internet]. 2014 [cited 2014 Jul 27];15:410. Available from: http://genomebiology.com/2014/15/7/410
13. Liao Y, Smyth GK, Shi W. featureCounts: an efficient general purpose program for assigning sequence reads to genomic features. Bioinformatics [Internet]. 2014 [cited 2015 Jan 12];30:923–30. Available from: http://www.ncbi.nlm.nih.gov/pubmed/24227677
14. Suárez-Vega A, Gutiérrez-Gil B, Klopp C, Tosser-Klopp G, Arranz J-J. Comprehensive RNA-Seq profiling to evaluate lactating sheep mammary gland

transcriptome. Sci. Data [Internet]. Nature Publishing Group; 2016 [cited 2017 Sep 25];3:160051. Available from: http://www.nature.com/articles/sdata201651
15. Robinson M, McCarthy D, Smyth G. edgeR: a Bioconductor package for differential expression analysis of digital gene expression data. Bioinformatics [Internet]. 2010;26:139–40. Available from: http://dx.doi.org/10.1093/bioinformatics/btp616

Personal Cover Click here to download Personal Cover Cover letter for review.docx

Dear Editors,

Here we present our manuscript entitled "Necklace: combining reference and assembled transcriptomes for more comprehensive RNA-Seq analysis" for your consideration as a Technical Note in Gigascience.

The best way to analyse RNA-Seq data in organisms with a poorly assembled or annotated genome has been a long standing issue. In such cases, analysis generally involves one of three approaches: relying on the reference genome and annotation, performing a genome-guided transcriptome assembly or performing de novo transcriptome assembly. Each of these approaches has their own benefits and limitations, therefore it is common for multiple approaches to be combined.

Despite the complementary information contained in reference and assembled transcriptomes integration of these has rarely been attempted and has been at best *ad hoc.* The approaches have been specific to the data set studied and ultimately difficult to reproduce. To the best of our knowledge, no software has existed for this task. In this manuscript we aim to rectify this with necklace, a pipeline which automates the steps from assembly through to read counting, in order to improve gene-models for any organism with a reference genome. Our approach is based on a recently published idea called "superTranscripts" (Davidson, Hawkins and Oshlack, Genome Bioloigy, August 2017), which has received a lot of attention in the research community (Altmetric Attention Score in the top 3% of research output the same age).

In the submitted manuscript, we describe the necklace pipeline and provide an example of how it improves a sheep milk differential expression analysis. Sheep has a fairly well assembled and annotated genome compared to many other organisms however necklace is able to identify more than 2000 additional genes with 66 of these being differentially expressed in the data set. We expect necklace to be a valuable tool for a broad range of species, making the tool of interest to a large section of the bioinformatics community.

We thank you for your consideration of our manuscript.

Kind regards,

Nadia Davidson and Alicia Oshlack
