# Supplementary material for: Necklace: combining reference and assembled transcriptomes for more comprehensive RNA-Seq analysis
Source: Gigascience. 2018 May 2;7(5):giy045. doi: 10.1093/gigascience/giy045 (PMC5946861; doi:10.1093/gigascience/giy045)
Supplement: GIGA-D-17-00354_R1.pdf [file giy045_giga-d-17-00354_r1.pdf]

GigaScience

Necklace: combining reference and assembled transcriptomes for more comprehensive RNA-Seq analysis

--Manuscript Draft--

**Manuscript Number:**	GIGA-D-17-00354R1
**Full Title:**	Necklace: combining reference and assembled transcriptomes for more comprehensive RNA-Seq analysis
**Article Type:**	Technical Note
**Funding Information:**	National Health and Medical Research Council (GNT1126157) — Prof. Alicia Oshlack
**Abstract:**	Background: RNA-Seq analyses can benefit from performing a genome-guided and de novo assembly, in particular for species where the reference genome or the annotation is incomplete. However, tools for integrating assembled transcriptome with reference annotation are lacking.<br>Findings: Necklace is a software pipeline that runs genome-guided and de novo assembly and combines the resulting transcriptomes with reference genome annotations. Necklace constructs a compact but comprehensive superTranscriptome out of the assembled and reference data. Reads are subsequently aligned and counted in preparation for differential expression testing.<br>Conclusions: Necklace allows a comprehensive transcriptome to be built from a combination of assembled and annotated transcripts which results in a more comprehensive transcriptome for the majority of organisms. In addition RNA-seq data is mapped back to this newly created superTranscript reference to enable differential expression testing with standard methods. Necklace is available from https://github.com/Oshlack/necklace/wiki under GPL 3.0.
**Corresponding Author:**	Alicia Oshlack<br><br>AUSTRALIA
**Corresponding Author Secondary Information:**	
**Corresponding Author's Institution:**	
**Corresponding Author's Secondary Institution:**	
**First Author:**	Nadia Davidson
**First Author Secondary Information:**	
**Order of Authors:**	Nadia Davidson<br>Alicia Oshlack
**Order of Authors Secondary Information:**	
**Response to Reviewers:**	Editor comments:<br><br>In addition, please register any new software application in the SciCrunch.org database to receive a RRID (Research Resource Identification Initiative ID) number, and include this in your manuscript. This will facilitate tracking, reproducibility and re-use of your tool.<br><br>We have now registered Necklace with SciCrunch, RRID SCR_016103 and quote this ID in the conclusion of the manuscript.<br><br><br>Reviewer #1:<br><br>1. What's the purpose of the second stage "Clustering of transcripts"? Since the

Powered by Editorial Manager® and ProduXion Manager® from Aries Systems Corporation

program Lace also has a clustering step with "Corset", the "Clustering of transcripts" stage seems redundant. Though the authors mentioned two benefits (add annotation information and filter wrong transcripts) of this stage, these two benefits can be achieved in some post-processing step.

Corset and Lace are separate programs, and only Lace is used in Necklace. Although Corset could be used for clustering prior to running Lace, we instead used a custom script that parses blat results and is tailored to accurately cluster cross-species transcripts.

The authors says Necklace assigns the de novo assembled transcript to the gene cluster, but in Figure 1 there is an arrow from "genome-based superTranscriptome" to this stage. I'm confused by the terms here. Does the "de novo assembled transcripts" here means only Trinity's output or the results from both StringTie and Trinity?

In the manuscript we refer to de novo assembled transcripts as being the contigs produced from Trinity assembly and genome guided assembly being transcripts assembled from StringTie. The clustering step involves assigning each de novo assembled transcript to a "genome-based superTranscript" from either the species being studied, or the related species. This step requires as input the genome-based superTranscriptome as well as the de novo assembly (as we align the de novo assembly against these). We represent this input relationship with the arrows in Figure 1. Genome-guided transcripts from StringTie are assigned to genes based on their position in the genome prior to building the genome-based superTranscriptome during the “Assembly” stage. In the “Reassembly” stage we only require the output of the clustering stage so we have updated the figure to remove the other arrows as suggested.

2. In the application, the authors gave the example of the improved of a gene where the reference genome is incomplete. I'm wondering how Necklace compares against this naive workflow:

(1) For each transcript from StringTie's output, extract its sequence (concatenate the sequences of its exons).

(2) Put the sequences from StringTie and sequences from Trinity together, and run Lace on this bigger set of sequences.

In other words, this naive workflow is just a simple application of Lace. And this is similar to the section "Combining reference and de novo assembled transcriptome" from superTranscript's paper except that there is no annotation here. The comparison of Necklace and this naive workflow would be much fairer.

To clarify, Necklace uses the same workflow (with some minor improvements) as the one presented in the "Combining reference and de novo assembled transcriptome" section of our superTranscript paper. The main goal of this manuscript is to present the software which automates that workflow in a user friendly way. Executing the workflow described by the reviewer could potentially be challenging for a non-expert and indeed automating these steps is likely to give a pipeline very similar to Necklace. The main differences between the review’s purposed pipeline and Necklace are:
-Necklace also uses reference annotation. Reference annotation will improve the gene-models, it allows novel transcripts to be annotated and its inclusion is a minor extension to the pipeline.
-Necklace builds a genome-based superTranscriptome. This step should not alter the completeness or correctness of the annotation, but is a convenience: 1) it reduces the amount of sequence that needs to be processed in the clustering and Lace steps, and 2) It ensures that Lace will output exons in genomic order. We have now added a statement to clarify this in the manuscript subsection “Assembly”.
-Necklace uses purpose built clustering rather than Corset and only accepts novel genes which match a protein coding gene in the related species. This point also relates to review #2’s comment 4. We now also address this point it in the manuscript in the sections: “Clustering of transcripts”, “Application to…” and “Conclusion.

Powered by Editorial Manager® and ProduXion Manager® from Aries Systems Corporation

1. The pipeline downloads all the required software packages regardless of whether they exist in the system. As a result, the pipeline alone takes about 3G disk space. Can the user specify the path to already-installed software so that Necklace could directly use it?

Yes, it is possible to specify already installed software in Necklace's file, "tools.groovy". We have now made this clearer in our Installation documentation on our github wiki.

2. Is there a way to set some environment variable for Necklace? For example, on my server the default Java version is v1.7 while I can direct Java to other versions such as v1.8. The Trinity requires Java version at least 1.8, and even though I specify Java to v1.8 on my server but bpipe
still use the default one. I got around this problem by change Trinity's script to access the desired Java version. Can this be resolved in an easier fashion?

Because Trinity requires an environment with java version 1.8, Necklace also requires this version of java. A work around is now available as de novo assembly can be run outside of Necklace, for example with a different assembler that does not require java 1.8. as requested below. We also provide this suggestion on our new wiki section, "FAQ".

3. The software for alignment and transcriptome assembly is rapidly evolving. Will Necklace allow the users to use their own choice of software? For example, they can use Shannon over Trinity.

This is an excellent point. Because tools don't tend to use the same arguments, and input and output formats, it would be difficult to make Necklace work with any generic assembler in a user friendly way. However, we acknowledge that users may want to use alternative software for their assemblies for a variety of reasons. Therefore we have now included an option whereby an assembly performed outside of Necklace (either de novo or genome-guided) can be passed to the pipeline and the default assembly is bypassed.

Minor comments:

On page 4 line 31 and on page 5, line 52, the first letter of "Necklace" should be "n" to be consistent, though I think the first letter of the software should always be capitalized.

On page 5, line 52, the "c/c++" should be capitalized to "C/C++".

On page 7, line 40, "stringTie" should be "StringTie".

We have now corrected these errors.

Reviewer #2:

Considering the vast majority of sequenced organisms with an incomplete and poorly annotated genome, I do think that Necklace would be of particular interest for a wide range of users. However, my main concern is related to the availability of Necklace, which is only developed for Linux. Being a Mac OS X user, I had to modify the source code of Necklace to install it on my own device. While the tools provided with their source code (cluster, make_blocks, samtools) were successfully compiled during the installation process, I had to modify the web links for the other tools (HISAT2, StringTie, featureCounts) to download the appropriate binaries since they are available for both Linux and Mac OS X platforms. The only tool that remained challenging was Trinity, which has been deprecated from Homebrew and needs to be installed by using Docker. As I did not manage to associate the execution of Trinity on the Docker with Necklace, I had to run them separately and modify the source code of the file "de_novo_assembly.groovy" to link the Trinity output file. Therefore, I suggest that Necklace would be also implemented on Mac OS X platform, so that a wider range of users could benefit from it.

We now provide an installation script for MacOS which has been tested. The

Powered by Editorial Manager® and ProduXion Manager® from Aries Systems Corporation

installation instructions on our wiki have also been modified.

Specific comments related to Necklace:

1) Linked with what I previously mentioned, since Trinity requires a lot of computer resources to run, I wonder whether an option should be provided to bypass Trinity execution. In this case, users with limited computation resources could run Trinity on external servers, such as Galaxy, and then provide the Trinity output file directly to Necklace.

We have now implemented this option.

2) I think that the list of input parameters that one can provide to Necklace is too limited. Currently, one can only adjust the number of threads, the maximum memory allocated to Trinity and the score/identity thresholds for Blat alignment to build the superTranscriptome. For instances, indicating the strand specificity to HISAT2, StingTie and Trinity, or restricting the fragment counting to pairs with both reads mapped (featureCounts -B), would be more appropriate in certain cases. Given that these tools accept numerous parameters, the simplest and easiest way may be to allow users to provide a string with all wanted parameters and arguments for a given tool, thus preventing them to modify the Necklace source code to adapt their analysis. Another alternative could be to include a file of parameters that advanced users can modify at their own appreciation.

This is a great suggestion and we now provide command line options to pass various strings to the tools used by Necklace. These are documented at https://github.com/Oshlack/necklace/wiki/Options

3) Currently, the genome and annotation(s) of only one related species can be provided to Necklace. Could the authors modify this so that users can provide information from several related species? As compared to mammals, yeasts and flies, there is no bird species with an accurate genome sequence and annotation. Therefore, I think that performing the comparison of newly annotated gene candidates to a single related species may be too restrictive.

We thank the reviewer for this suggestion. We believe this can be addressed by running Necklace iteratively to include information from multiple species. We have now updated our documentation to explain this application (see the new FAQ section of the wiki).

4) Although I completely understand the strategy employed by the authors during the transcript clustering, I not sure whether this is the most appropriate approach. On one hand, candidate genes generated from de novo assembly must match either a gene detected during the genome-guided superTranscriptome, or a gene present in the related species superTranscriptome. This may result in the loss of a significant proportion of gene candidates if the studied organism has an incomplete genome and the related species is poorly annotated or too distant to be efficiently compared at the nucleotide level. For example, it has been shown that genes encoding long non-coding RNAs have rapidly diverged through evolution as compared to protein-coding genes. On the other hand, the authors mentioned that de novo assembled transcripts mapped on more than one gene are also removed. Genomes that contain numerous gaps and unassigned contigs (e.g. chicken) tend to display a non-negligible proportion of fragmented genes, i.e. spanning on multiple contigs, of which each region is erroneously annotated as a distinct gene. Thus, de novo assembled transcripts spanning on several regions of a fragmented gene would be wrongly defined as false chimera and be subsequently ignored. Could the authors discuss these two points?

This is a valid concern and we accept that our approach may miss novel transcribed sequence and/or be unable to reconstruct “fragmented” genes, which are two of the strengths of de novo transcriptome assembly. However, very often the assembled contigs that are inconsistent or absent from both the reference genome and a related species, are assembly errors. For example, false chimeras are an extremely common assembly artefact that give the false appearance of a “fragmented” gene. In Necklace we have intentionally made the decision to prioritise correctness over completeness

	and we still manage to recover novel genes in all our example data sets. In addition we have added a discussion of this point in the text in the sections: “Clustering of transcripts” and “Conclusion”.<br><br>Specific comments for the manuscript:<br><br>1) I suggest to the authors to further expand the current limitations of RNA-seq analysis on poorly annotated organisms in the introduction. This would help the non-specialist readers to clearly identify the usefulness of Necklace for their RNA-seq data analysis.<br><br>We have now expanded the introduction with this suggestion.<br><br>2) Figure 1: the arrowhead spanning from the "de novo assembly" box to the clustering is hidden. Could the authors also highlight differently tools and file formats? Both are in bold.<br><br>We have corrected this.<br><br>3) Page 4, line 18: "superTrascriptome" should be replaced by "superTranscriptome".<br><br>We have corrected this.<br><br>4) Could the authors describe the current version of the Churra sheep milk genome (Oar_v3.1) that they used for validation? In terms of genome size, number of gaps, number of sequenced chromosomes, number of unassigned contigs<br><br>We have added this information to the manuscript in the section “Application to differential expression testing in sheep transcriptomes”.<br><br>5) Could the authors give more details on their results with the sheep transcriptome? Could they assign a function to the 2,208 additional genes identified and to the 66 differentially expressed genes newly detected? Are they known protein-coding genes? Do they encode putative proteins or non-coding RNAs? This would highlight the potential benefits of running Necklace on partially annotated genomes. I also suggest to the authors to include a table summarizing the results, in order to help the reader.<br><br>We now include these details and provide a summary table.<br><br>6) Page 5, lines 6 and 7 (as well as legend of figure 2): "321bp" and "3333bp" should be written "321 bp" and "3333 bp", respectively.<br><br>We have corrected this.<br><br>7) I think authors should expand further in their conclusions what are the strengths of Necklace. The idea of combining genome-guided and de novo assemblies prior to RNA-seq analysis is not new, but Necklace is the first automated pipeline to my knowledge that is user-friendly, allows a high reproducibility and seems adaptable to a larger scale. However, it is also noteworthy to mention the potential limitations encountered when analysing RNA-seq data on highly fragmented genomes or from organisms with no closely-related species with accurate gene annotation.<br><br>We have now expanded the Conclusions section with these suggestions.<br><br>8) In the methods section, could the authors indicate the time of execution for the different commands?<br><br>We now include this information.<br><br>List of abbreviations (page 8): "fragements" should be corrected to "fragments".<br><br>We have corrected this.
**Additional Information:**	

Powered by Editorial Manager® and ProduXion Manager® from Aries Systems Corporation

Question	Response
Are you submitting this manuscript to a special series or article collection?	No
**Experimental design and statistics**<br><br>Full details of the experimental design and statistical methods used should be given in the Methods section, as detailed in our Minimum Standards Reporting Checklist. Information essential to interpreting the data presented should be made available in the figure legends.<br><br>Have you included all the information requested in your manuscript?	Yes
**Resources**<br><br>A description of all resources used, including antibodies, cell lines, animals and software tools, with enough information to allow them to be uniquely identified, should be included in the Methods section. Authors are strongly encouraged to cite Research Resource Identifiers (RRIDs) for antibodies, model organisms and tools, where possible.<br><br>Have you included the information requested as detailed in our Minimum Standards Reporting Checklist?	Yes
**Availability of data and materials**<br><br>All datasets and code on which the conclusions of the paper rely must be either included in your submission or deposited in publicly available repositories (where available and ethically appropriate), referencing such data using a unique identifier in the references and in the "Availability of Data and Materials" section of your manuscript.<br><br>Have you have met the above requirement as detailed in our Minimum Standards Reporting Checklist?	Yes

Manuscript
Click here to download Manuscript Necklace Gigascience_r3.docx

Necklace: combining reference and assembled transcriptomes for more comprehensive RNA-Seq analysis

Nadia M Davidson[1,2,*] and Alicia Oshlack[1,2,*]
[1]Murdoch Childrens Research Institute, Royal Children's Hospital, Victoria, Australia
[2]School of Bio-Sciences, University of Melbourne, Victoria, Australia
*To whom correspondence should be addressed.
Contact: nadia.davidson@mcri.edu.au or alicia.oshlack@mcri.edu.au

Abstract
Background: RNA-Seq analyses can benefit from performing a genome-guided and de novo assembly, in particular for species where the reference genome or the annotation is incomplete. However, tools for integrating assembled transcriptome with reference annotation are lacking.
Findings: Necklace is a software pipeline that runs genome-guided and de novo assembly and combines the resulting transcriptomes with reference genome annotations. Necklace constructs a compact but comprehensive superTranscriptome out of the assembled and reference data. Reads are subsequently aligned and counted in preparation for differential expression testing.
Conclusions: Necklace allows a comprehensive transcriptome to be built from a combination of assembled and annotated transcripts which results in a more comprehensive transcriptome for the majority of organisms. In addition RNA-seq data is mapped back to this newly created superTranscript reference to enable differential expression testing with standard methods. Necklace is available from https://github.com/Oshlack/necklace/wiki under GPL 3.0.

Keywords: transcriptome, assembly, RNA-Seq, non-model

Findings

Introduction
Despite the increasing number of species with a sequenced genome, the vast majority of reference genomes are incomplete. They may contain gaps, have unplaced assembly scaffolds and be poorly annotated. The naïve approach to analysing RNA-Seq on species with a genome would follow the same procedure as model organisms; align reads the genome and count reads overlapping annotated genes, then test for differential expression based on gene counts [1]. However, this approach has the potential to miss important biology for many organisms. Segments of genes may be missed, either because of a gap in the reference sequence or missing annotation. The downstream differential expression analysis is likely to have reduced statistical power because the gene counts are underestimated. Similarly, we have observed different segments of a gene being annotated as separate genes such as when the gene spans multiple

assembly scaffolds. However, this can happen even when a gene sits within a single scaffold. In the worst case, whole genes can be missed.

Ideally, an RNA-Seq analysis could repair the gene-models available from a reference genome and annotation, by extracting information about the expressed genes from the data itself through genome-guided and/or de novo assembly [2]. However, analyses involving assembly remain complex, more so when multiple assemblies need to be integrated. Prior works such as [3] have gone some way to addressing the challenge, however no reusable software has been written to perform these types of analyses.

In Davidson et al. 2017, [4] we introduced the concept of the superTranscriptome, where each gene is represented by one sequence containing all of that gene's exons in transcriptional order. SuperTranscripts provided a convenient means in which transcriptomes from difference sources, such as assembly and annotation, can be combined into a compact and unified reference. When applied to chicken, we showed that we could recover hundreds of segments of genes that were absent from the chicken reference genome.

Here we present software called Necklace which automates the process described in [4] for any species with an incomplete reference genome. Necklace takes as input a configuration file containing paths to the RNA-seq reads, a reference genome and one or more reference genome annotation. Because de novo assembly is error prone, we require that any gene discovered specifically through de novo assembly be also found amongst the coding sequence of a related (well annotated) species. Therefore, the genome and annotation of a related species must also be provided to Necklace. Necklace will then run the steps involved in genome-guided and de novo assembly, and combine the assembled transcriptome with reference annotations for the species of interest. After building the superTranscriptome, Necklace will align and count reads in preparation for testing for differential gene expression and differential transcript usage using well established tools such as edgeR [5], DEseq[6] or DEXseq[7].

In order to demonstrate the application of Necklace in a new data set we analysed public RNA-seq data from sheep milk. Compared to using the sheep reference genome on it own, the Necklace analysis resulted in 18% more reads being assigned to genes and 19% more differentially expressed genes being detected.

The Necklace pipeline

Necklace is a pipeline constructed using the bpipe framework [8]. It steers external software, such as aligners and assemblers, as well as a set of its own utilities, written in C/C++. As input Necklace takes the raw RNA-seq reads and the reference genome for the species as well as any available annotation. In addition, it takes a reference genome and annotation from a related, but well studied species such as human, drosophila, yeast, etc. Necklace consists of several sequential stages: initial genome guided and *de novo* assembly, clustering transcripts into gene groupings, reassembly to build the superTranscriptome

and finally alignment and counting of mapped reads in preparation for differential expression testing and differential isoform usage testing. Each of these sequential stages consists of several sub-stages and is outlined in Figure 1 with further detail below.

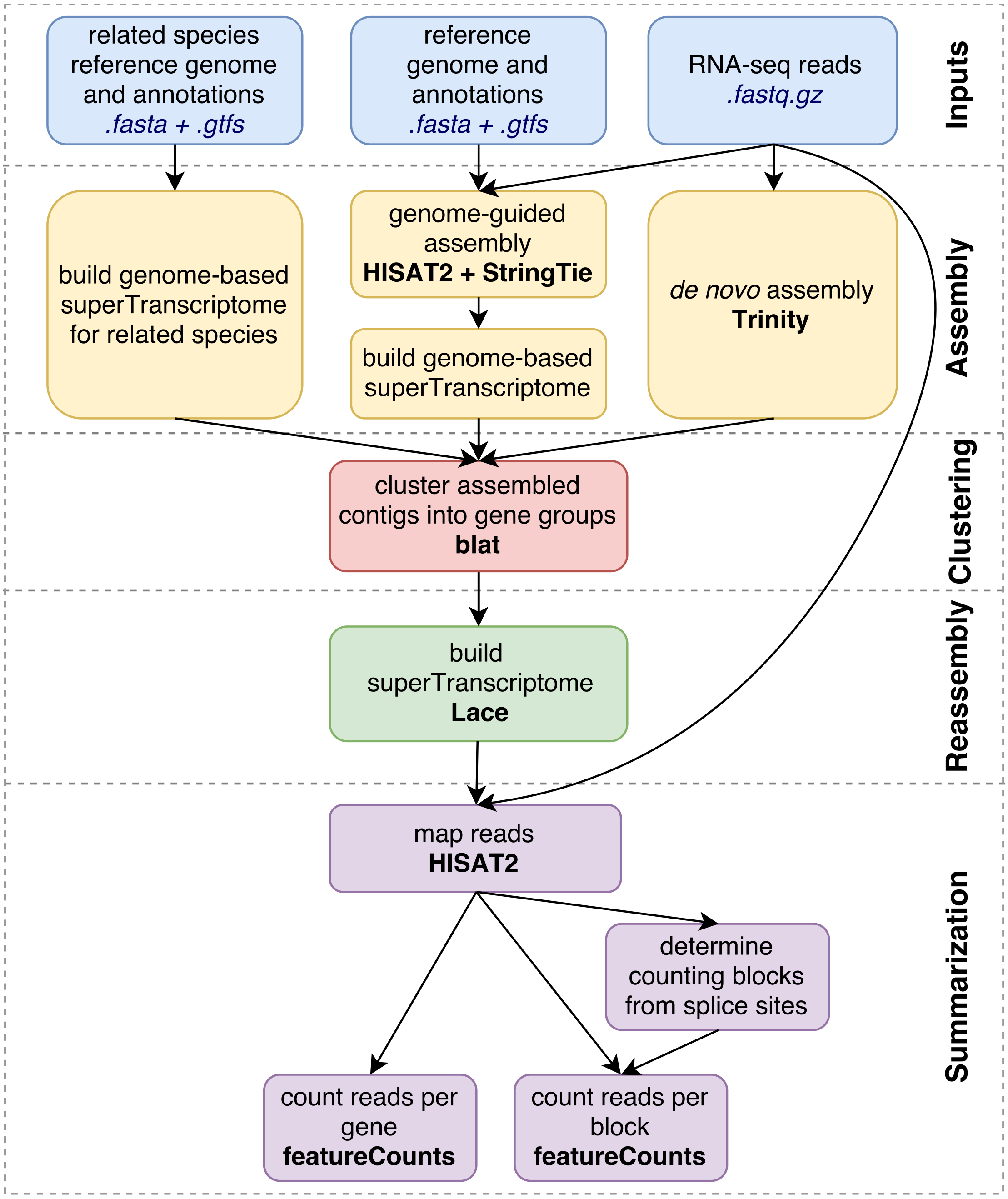

Figure 1. *An overview of the Necklace pipeline. External software that Necklace runs is shown in bold.*

Assembly

The assembly stage creates three different transcriptomes. First reads are aligned to the reference genome using HISAT2 [9] and genome-guided assembly is performed with StringTie [10]. This assembly is combined with reference annotations and then flattened based on genomic location, so that each exon is

reported only once and overlapping exons are merged. Exonic sequence is then extracted from the genome and concatenated to build a "genome-based" superTranscriptome. We build the initial genome-based superTranscriptome rather than use a set of transcript sequences for two reasons. Firstly, it results in the correct genomic order of a gene's exons. This ensures that when Lace is used in the reassembly step to combine the de novo assembled transcripts, the final superTranscriptome also has exons in the correct genomic order. Secondly, this step reduces the amount of sequence to be processed in the Clustering and Reassembly Steps.

In parallel to building the genome-based superTranscriptome for the species of interest, the related species annotation is used to create a genome-based superTranscriptome (without genome-guided assembly). Finally, RNA-Seq reads are de novo assembled with Trinity [11].

Clustering of transcripts

This step assigns de novo assembled transcripts to gene clusters prior to building the final superTranscriptome. Those contigs aligning to the genome-based superTranscriptome (using Blat [12]) are allocated to known genes while those not aligning to the genome, but found in the related species superTranscriptome are assigned to novel genes. De novo assembled transcripts that align to more than one gene are removed to avoid false chimeras [13] from being introduced into the superTranscriptome.

A limitation of this filtering is that de novo assembled contigs cannot be used to scaffold highly fragmented references because those contigs will give the appearance of false chimeras. Novel genes which are absent from the genome and the related species will also be missed with our approach. However, de novo assemblies are highly error prone and strict filtering of assembled transcripts is required to eliminate the introduction of artifacts into the annotation.

Reassembly of superTranscripts

Each cluster consists of a gene's genome-based superTranscript and/or its set of de novo assembled transcripts. The transcripts in each cluster are merged together through Lace assembly [4], to produce one superTranscript per gene.

Summarization

Reads are aligned back to the superTranscriptome using HISAT2 and fragments counted per gene using featureCounts [14]. Splice junctions reported by HISAT2 are used to segment each superTranscript into a set of contiguous "blocks". Fragments are then counted in "blocks" and can be used for differential isoform detection like exon counts.

Application to differential expression testing in sheep transcriptomes

To demonstrate the utility of necklace, we applied it to public RNA-Seq from Churra sheep milk and compared transcriptome expression at day 10 to day 150 post lambing [15]. Necklace was given the sheep reference genome, Oar_v3.1. This version of the sheep genome is 2.6 GB in size, with 85 MB of unfilled

assembly gaps. It consists of 5,698 scaffolds (28 chromosomes and 5,670 unplaced contigs). Human, with the hg38 reference genome, was used as the related species. For both genomes, version 90 of the Ensembl annotation was used (see methods).

The Ensembl reference consisted of 29,118 transcripts and reference guided assembly using StringTie resulted in 65,717 transcripts. The sheep data was *de novo* assembled into 267,553 contigs, however only 63,592 contigs were reassembled into the Necklace superTranscriptome due to filtering at the clustering step. The magnitude of this reduction is consistent with alternative clustering methods (e.g. the removal of contigs with little read support using Corset [13]).

Using this data and set of reference files resulted in a more comprehensive transcriptome. Compared to the Ensembl sheep annotation, the number of bases included in the Necklace transcriptome increased by 76% and 18% more reads were assigned to genes by featureCounts (Table 1). This more comprehensive reference included 2208 (8%) more genes. Of the novel genes, 1303 were found to have an open reading frame of 100 or more amino acids and 587 of these had homology to protein sequence from another species.

When performing differential expression analysis using edgeR the necklace transcriptome identified more significantly differentially expressed genes than using the reference alone (456 compared to 383, FDR<0.05). Some of these differences could be attributed to the inclusion of novel unannotated genes, with 66 of the newly annotated genes identified as differentially expressed. A protein coding homology could be found for 21 of the novel differentially expressed genes. Necklace was also able to improve the detection of differential expression amongst several known genes by providing more complete gene sequences. Larger numbers of reads mapping to the longer sequences resulted in more power for differential expression testing. One example of this was the *SERTM1* gene where the annotated transcript only included 321 bp while the necklace superTranscript contained 3333 bp and overlapped a genome assembly gap (Figure 2).

	**Reference**	**Necklace**
**Bases (Mbp)**	45.13	79.56
**Reads assigned to genes**	194,693,051	230,140,801
**Genes**	26,613	28,821
**Differentially expressed genes**	383	456

Table 1. *A comparison of using the Ensembl reference annotation alone and using the superTranscriptome generated by Necklace for our example sheep dataset.*

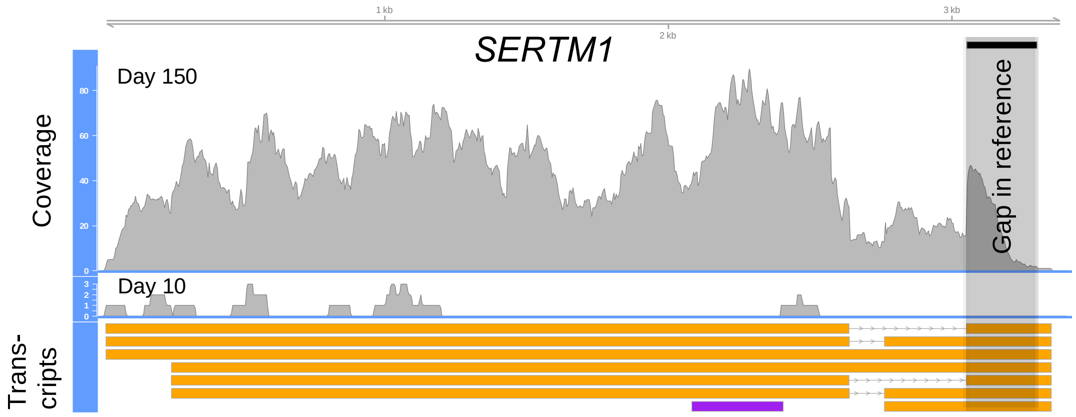

Figure 2. *Read coverage aggregated over replicate samples for the Necklace assembled superTranscript of SERTM1. This gene is found to be significantly differentially expressed using the Necklace generated reference, but is missed when the reference genome and annotation are used in isolation due to low read counts. The reference annotation consists of a single transcript of 321 bp (shown in purple), whereas the de novo assembled gene consists of seven transcripts up to 3333 bp long (shown in orange) and includes approximately 250bp that is absent from the reference genome, in a location consistent with a genome assembly gap. The genome-guided transcripts that were assembled for this gene were filtered out by StringTie's merge function due to an average FPKM < 1.*

Conclusion

Here we have presented necklace, a pipeline designed to improve RNA-seq analysis in species with an incomplete genome and annotation. We believe necklace is the first pipeline to automate the steps required to combine reference and assembled data, alignment and summarization of counts thereby making the analysis process user friendly and reproducible.

Incorporating *de novo* assembled data into the analysis of species with a semi-complete genome promises to give a more comprehensive picture of the transcriptome. However, de novo assemblies can also introduce artifacts and for this reason Necklace only adds de novo assembled transcripts corresponding to novel protein coding genes which are found in a related species. Assembled transcripts that match multiple known genes are also removed, in order to prevent the introduction of false chimeric sequences. Therefore it is still possible that some *bona fide* expressed transcripts are not included in the final results.

In real data, we show that indeed applying Necklace to a sheep data set resulted in a more complete transcriptome. We were able to discover more exon and

more genes, some of which had homology to known protein coding sequences in other species. This analysis also resulted in more power for differential expression analysis. Necklace is open source and available from github at https://github.com/Oshlack/necklace/wiki and has resource RRID:SCR_016103.

Methods

Data

Sheep RNA-Seq data was downloaded from SRA (accession numbers SRR2932539- SRR2932542,SRR2932561-SRR2932564). The sheep genome and annotation was downloaded from Ensembl:
ftp://ftp.ensembl.org/pub/release-90/fasta/ovis_aries/dna/Ovis_aries.Oar_v3.1.dna.toplevel.fa.gz
ftp://ftp.ensembl.org/pub/release-90/gtf/ovis_aries/Ovis_aries.Oar_v3.1.90.gtf.gz

The human reference genome and annotation was also downloaded from Ensembl:
ftp://ftp.ensembl.org/pub/release-90/fasta/homo_sapiens/dna/Homo_sapiens.GRCh38.dna.toplevel.fa.gz,
ftp://ftp.ensembl.org/pub/release-90/gtf/homo_sapiens/Homo_sapiens.GRCh38.90.gtf.gz

We then selected coding sequence from the human annotation using the command:
```
grep "	CDS	" data/Homo_sapiens.GRCh38.90.gtf > Homo_sapiens.GRCh38.90.CDS.gtf
```

Necklace Analysis

For the necklace analysis of sheep milk, all data files were placed into a subdirectory called “data” and a necklace input file, “data.txt”, was created with the following lines:

```
// sequencing data
reads_R1="data/SRR2932539_1.fastq.gz,data/SRR2932540_1.fastq.gz,data/SRR2932541_1.fastq.gz,data/SRR2932542_1.fastq.gz,data/SRR2932561_1.fastq.gz,data/SRR2932562_1.fastq.gz,data/SRR2932563_1.fastq.gz,data/SRR2932564_1.fastq.gz"
reads_R2="data/SRR2932539_2.fastq.gz,data/SRR2932540_2.fastq.gz,data/SRR2932541_2.fastq.gz,data/SRR2932542_2.fastq.gz,data/SRR2932561_2.fastq.gz,data/SRR2932562_2.fastq.gz,data/SRR2932563_2.fastq.gz,data/SRR2932564_2.fastq.gz"

//The genome and annotation
annotation="data/Ovis_aries.Oar_v3.1.90.gtf"
genome="data/Ovis_aries.Oar_v3.1.dna.toplevel.fa"
```

```
//The genome and annotation of a related species
annotation_related_species="data/Homo_sapiens.GRCh38.90.CDS.gtf"
genome_related_species="data/Homo_sapiens.GRCh38.dna.toplevel.fa"
```

Necklace version 0.9 was then run using the command:

```
<necklace path>/tools/bin/bpipe run -n 8 <necklace path>/necklace.groovy
data/data.txt
```

Version numbers of all the external tools that necklace calls can be found in necklace's installation script, "install_linux64.sh".

Necklace was run on 8 cores on a 48 core AMD Opteron(tm) Processor 6180 SE 2.5 GHz CentOS 6.7 server with 252GB of RAM. The full run time was approximately 4.5 days.

The assembly stage took approximately 3.5 days due to the *de novo* assembly (Trinity was run with 6 threads and 50 GB memory maximum). Genome-guided assembly was run concurrently on 1 core and took 20 hours. The time for clustering was 11 hours, which was dominated by alignment of the *de novo* assembled contigs to the related species with blat. Lace ran in 13 hours and read realignment and summarization took 1.5 hours.

Annotation of Novel Genes

StringTie and Trinity sequence from novel sheep genes were extracted and analysed with TransDecode [16]and blastp [17] against the UniProt [18] database using the commands:

```
TransDecoder.LongOrfs -t <transcripts.fasta>

blastp -query transdecoder_dir/longest_orfs.pep  \
   -db uniprot_sprot.fasta  -max_target_seqs 1 \
   -outfmt 6 -evalue 1e-5 -num_threads 10 > blastp.outfmt6

hmmscan --cpu 8 --domtblout pfam.domtblout /path/to/Pfam-A.hmm
transdecoder_dir/longest_orfs.pep

TransDecoder.Predict -t target_transcripts.fasta --retain_pfam_hits
pfam.domtblout --retain_blastp_hits blastp.outfmt6
```

Reference Based Analysis

To make the reference based analysis as similar as possible to the necklace pipeline we used the versions of HISAT2, samtools and featureCounts that were installed by necklace.

HISAT2 was run on each sample using the command:

```
hisat2 --known-splicesite-infile <splice sites file> -x <genome index> -1
<input_1.fastq.gz> -2 <input_2.fastq.gz> | samtools view -u - > <output.bam>
```

Where the splice sites file and genome index were the same ones generated in the initial stage of necklace that aligns reads to the reference genome.

Reads were then counted for each annotated gene using featureCounts with the command:

```
featureCounts -T 8 --primary -p -t exon -g gene_id -a Ovis_aries.Oar_v3.1.90.flat.gtf -o counts *.bam
```

Where "Ovis_aries.Oar_v3.1.90.flat.gtf" was a flattened version of the sheep Ensembl annotation and was created with the necklace command:

```
gtf2flatgtf Ovis_aries.Oar_v3.1.90.gtf Ovis_aries.Oar_v3.1.90.flat.gtf
```

Flattening the annotation involves merging transcripts of a gene into a non-redundant but complete set of exons.

Differential Expression Testing

For differential gene expression testing, gene-level counts were analysed using the R bioconductor package edgeR (version 3.18.1) [19]. We modeled both the time-point post lambing and animal in the design matrix:

```
time_point<-c(rep("Day10",4),rep("Day150",4))
indv<-c(3141,4860,49537,9539,3141,4860,9539,49537) //numbers are animal IDs
design <- model.matrix(~0+factor(indv)+factor(time_point))
colnames(design) <- gsub("factor","",colnames(design))
sample_names=paste(indv,time_point,sep="_")
rownames(design)=sample_names
```

The counts table was read into R and passed to edgeR:

```
counts=count_table[,7:ncol(count_table)]
y <- DGEList(counts=counts)
```

Genes with a counts per million (cpm) less than or equal to 0.5 in less 4 samples were filtered out and the libraries normalized.

```
keep <- rowSums(cpm(y) > 0.5) >=4
y <- y[keep, , keep.lib.sizes=TRUE]
y <- calcNormFactors(y)
```

We then estimated the dispersion and looked for differential expression with a false discovery rate (FDR) < 0.05:

```
y <- estimateDisp(y,design,robust=TRUE)
fit <- glmFit(y, design,robust=TRUE)
qlf <- glmLRT(fit,coef=5)
is.de <- decideTests(qlf, p.value=0.05)
```

Availability of supporting source code and requirements

Project name: Necklace

Project home page: https://github.com/Oshlack/necklace/wiki

Operating system(s): Linux

Programming language: Groovy and C/C++

Other requirements: Java 1.8

License: GPL 3.0

Declarations

List of abbreviations

FPKM – fragments per kilobase of exon per million mapped reads

Competing interests

None declared.

Funding

AO is funded by an *NHMRC CDF GNT1126157.*

Authors' contributions

ND wrote all the software and drafted the paper. AO oversaw the project and contributed to writing the manuscript.

Acknowledgements

We would like to thank Anthony Hawkins, the author of Lace, who contributed to the early concept of necklace when applied to chicken.

References

1. Oshlack A, Robinson MD, Young MD. From RNA-seq reads to differential expression results. Genome Biol. [Internet]. 2010 [cited 2013 Mar 2];11:220. Available from: http://genomebiology.com/2010/11/12/220
2. Martin J, Wang Z. Next-generation transcriptome assembly. Nat. Rev. Genet. [Internet]. 2011;12:671–82. Available from: http://dx.doi.org/10.1038/nrg3068
3. Orgeur M, Martens M, Börno ST, Timmermann B, Duprez D, Stricker S. A dual transcript-discovery approach to improve the delimitation of gene features from RNA-seq data in the chicken model. Biol. Open [Internet]. The Company of Biologists Ltd; 2017 [cited 2017 Dec 18];bio.028498. Available from: http://www.ncbi.nlm.nih.gov/pubmed/29183907
4. Davidson NM, Hawkins ADK, Oshlack A. SuperTranscripts: a data driven reference for analysis and visualisation of transcriptomes. Genome Biol. 2017 181 [Internet]. BioMed Central; 2017 [cited 2017 Sep 18];18:148. Available

from: https://genomebiology.biomedcentral.com/articles/10.1186/s13059-017-1284-1
5. Robinson M, McCarthy D, Chen Y, Smyth GK. edgeR: differential expression analysis of digital gene expression data User→s Guide. 2011;
6. Anders S, Huber W. Differential expression analysis for sequence count data. Genome Biol. [Internet]. 2010 [cited 2013 May 21];11:R106. Available from: http://genomebiology.com/2010/11/10/R106
7. Anders S, Reyes A, Huber W. Detecting differential usage of exons from RNA-seq data. Genome Res. [Internet]. Cold Spring Harbor Laboratory Press; 2012 [cited 2016 Sep 14];22:2008–17. Available from: http://www.ncbi.nlm.nih.gov/pubmed/22722343
8. Sadedin SP, Pope B, Oshlack A. Bpipe: a tool for running and managing bioinformatics pipelines. Bioinformatics [Internet]. 2012 [cited 2013 Nov 15];28:1525–6. Available from: http://bioinformatics.oxfordjournals.org/content/early/2012/04/11/bioinformatics.bts167.abstract
9. Kim D, Langmead B, Salzberg SL. HISAT: a fast spliced aligner with low memory requirements. Nat. Methods [Internet]. 2015 [cited 2016 Sep 14];12:357–60. Available from: http://www.ncbi.nlm.nih.gov/pubmed/25751142
10. Pertea M, Pertea GM, Antonescu CM, Chang T-C, Mendell JT, Salzberg SL. StringTie enables improved reconstruction of a transcriptome from RNA-seq reads. Nat. Biotechnol. [Internet]. Nature Research; 2015 [cited 2017 Sep 18];33:290. Available from: http://www.nature.com/nbt/journal/v33/n3/full/nbt.3122.html
11. Haas BJ, Papanicolaou A, Yassour M, Grabherr M, Blood PD, Bowden J, et al. De novo transcript sequence reconstruction from RNA-seq using the Trinity platform for reference generation and analysis. Nat. Protoc. [Internet]. Nature Publishing Group, a division of Macmillan Publishers Limited. All Rights Reserved.; 2013 [cited 2013 Oct 30];8:1494–512. Available from: http://dx.doi.org/10.1038/nprot.2013.084
12. Kent WJ. BLAT--the BLAST-like alignment tool. Genome Res. [Internet]. 2002 [cited 2013 May 29];12:656–64. Available from: http://www.pubmedcentral.nih.gov/articlerender.fcgi?artid=187518&tool=pmcentrez&rendertype=abstract
13. Davidson NM, Oshlack A. Corset: enabling differential gene expression analysis for de novo assembled transcriptomes. Genome Biol. [Internet]. 2014 [cited 2014 Jul 27];15:410. Available from: http://genomebiology.com/2014/15/7/410
14. Liao Y, Smyth GK, Shi W. featureCounts: an efficient general purpose program for assigning sequence reads to genomic features. Bioinformatics [Internet]. 2014 [cited 2015 Jan 12];30:923–30. Available from: http://www.ncbi.nlm.nih.gov/pubmed/24227677
15. Suárez-Vega A, Gutiérrez-Gil B, Klopp C, Tosser-Klopp G, Arranz J-J. Comprehensive RNA-Seq profiling to evaluate lactating sheep mammary gland transcriptome. Sci. Data [Internet]. Nature Publishing Group; 2016 [cited 2017 Sep 25];3:160051. Available from: http://www.nature.com/articles/sdata201651
16. Haas & Papanicolaou et al. TransDecoder (Find Coding Regions Within

Transcripts) [Internet]. Available from: https://github.com/TransDecoder/TransDecoder/wiki
17. McGinnis S, Madden T. BLAST: at the core of a powerful and diverse set of sequence analysis tools. Nucleic Acids Res. [Internet]. 2004;32:W20–5. Available from: http://dx.doi.org/10.1093/nar/gkh435
18. Bateman A, Martin MJ, O'Donovan C, Magrane M, Alpi E, Antunes R, et al. UniProt: the universal protein knowledgebase. Nucleic Acids Res. [Internet]. Oxford University Press; 2017 [cited 2018 Mar 2];45:D158–69. Available from: https://academic.oup.com/nar/article-lookup/doi/10.1093/nar/gkw1099
19. Robinson M, McCarthy D, Smyth G. edgeR: a Bioconductor package for differential expression analysis of digital gene expression data. Bioinformatics [Internet]. 2010;26:139–40. Available from: http://dx.doi.org/10.1093/bioinformatics/btp616

Personal Cover Click here to download Personal Cover reviews.docx

GigaScience GIGA-D-17-00354

Necklace: combining reference and assembled transcriptomes for more comprehensive RNA-Seq analysis

Nadia Davidson; Alicia Oshlack

Editor comments:

In addition, please register any new software application in the SciCrunch.org database to receive a RRID (Research Resource Identification Initiative ID) number, and include this in your manuscript. This will facilitate tracking, reproducibility and re-use of your tool.

We have now registered Necklace with SciCrunch, RRID SCR_016103 and quote this ID in the conclusion of the manuscript.

Reviewer #1:

1. What's the purpose of the second stage "Clustering of transcripts"? Since the program Lace also has a clustering step with "Corset", the "Clustering of transcripts" stage seems redundant. Though the authors mentioned two benefits (add annotation information and filter wrong transcripts) of this stage, these two benefits can be achieved in some post-processing step.

Corset and Lace are separate programs, and only Lace is used in Necklace. Although Corset could be used for clustering prior to running Lace, we instead used a custom script that parses blat results and is tailored to accurately cluster cross-species transcripts.

The authors says Necklace assigns the de novo assembled transcript to the gene cluster, but in Figure 1 there is an arrow from "genome-based superTranscriptome" to this stage. I'm confused by the terms here. Does the "de novo assembled transcripts" here means only Trinity's output or the results from both StringTie and Trinity?

In the manuscript we refer to de novo assembled transcripts as being the contigs produced from Trinity assembly and genome guided assembly being transcripts assembled from StringTie. The clustering step involves assigning each de novo assembled transcript to a "genome-based superTranscript" from either the species being studied, or the related species. This step requires as input the genome-based superTranscriptome as well as the de novo assembly (as we align the de novo assembly against these). We represent this input relationship with the arrows in Figure 1. Genome-guided transcripts from StringTie are assigned to genes based on their position in the genome prior to building the genome-based superTranscriptome during the "Assembly" stage. In the "Reassembly" stage we only require the output of the clustering stage so we have updated the figure to remove the other arrows as suggested.

2. In the application, the authors gave the example of the improved of a gene where the reference genome is incomplete. I'm wondering how Necklace compares against this naive workflow:

(1) For each transcript from StringTie's output, extract its sequence (concatenate the sequences of its exons).

(2) Put the sequences from StringTie and sequences from Trinity together, and run Lace on this bigger set of sequences.

In other words, this naive workflow is just a simple application of Lace. And this is similar to the section "Combining reference and de novo assembled transcriptome" from superTranscript's paper except that there is no annotation here. The comparison of Necklace and this naive workflow would be much fairer.

To clarify, Necklace uses the same workflow (with some minor improvements) as the one presented in the "Combining reference and de novo assembled transcriptome" section of our superTranscript paper. The main goal of this manuscript is to present the software which automates that workflow in a user friendly way. Executing the workflow described by the reviewer could potentially be challenging for a non-expert and indeed automating these steps is likely to give a pipeline very similar to Necklace. The main differences between the review's purposed pipeline and Necklace are:

- *Necklace also uses reference annotation. Reference annotation will improve the gene-models, it allows novel transcripts to be annotated and its inclusion is a minor extension to the pipeline.*
- *Necklace builds a genome-based superTranscriptome. This step should not alter the completeness or correctness of the annotation, but is a convenience: 1) it reduces the amount of sequence that needs to be processed in the clustering and Lace steps, and 2) It ensures that Lace will output exons in genomic order. We have now added a statement to clarify this in the manuscript subsection "Assembly".*
- *Necklace uses purpose built clustering rather than Corset and only accepts novel genes which match a protein coding gene in the related species. This point also relates to review #2's comment 4. We now also address this point it in the manuscript in the sections: "Clustering of transcripts", "Application to…" and "Conclusion.*

1. The pipeline downloads all the required software packages regardless of whether they exist in the system. As a result, the pipeline alone takes about 3G disk space. Can the user specify the path to already-installed software so that Necklace could directly use it?

Yes, it is possible to specify already installed software in Necklace's file, "tools.groovy". We have now made this clearer in our Installation documentation on our github wiki.

2. Is there a way to set some environment variable for Necklace? For example, on my server the default Java version is v1.7 while I can direct Java to other versions such as v1.8. The Trinity requires Java version at least 1.8, and even though I specify Java to v1.8 on my server but bpipe still use the default one. I got around this problem by change Trinity's script to access the desired Java version. Can this be resolved in an easier fashion?

Because Trinity requires an environment with java version 1.8, Necklace also requires this version of java. A work around is now available as de novo assembly can be run outside of Necklace, for example with a different assembler that does not require java 1.8. as requested below. We also provide this suggestion on our new wiki section, "FAQ".

3. The software for alignment and transcriptome assembly is rapidly evolving. Will Necklace allow the users to use their own choice of software? For example, they can use Shannon over Trinity.

This is an excellent point. Because tools don't tend to use the same arguments, and input and output formats, it would be difficult to make Necklace work with any generic assembler in a user friendly way. However, we acknowledge that users may want to use alternative software for their assemblies for a variety of reasons. Therefore we have now included an option whereby an assembly performed outside of Necklace (either de novo or genome-guided) can be passed to the pipeline and the default assembly is bypassed.

Minor comments:

On page 4 line 31 and on page 5, line 52, the first letter of "Necklace" should be "n" to be consistent, though I think the first letter of the software should always be capitalized.

On page 5, line 52, the "c/c++" should be capitalized to "C/C++".

On page 7, line 40, "stringTie" should be "StringTie".

We have now corrected these errors.

Reviewer #2:

Considering the vast majority of sequenced organisms with an incomplete and poorly annotated genome, I do think that Necklace would be of particular interest for a wide range of users. However, my main concern is related to the availability of Necklace, which is only developed for Linux. Being a Mac OS X user, I had to modify the source code of Necklace to install it on my own device. While the tools provided with their source code (cluster, make_blocks, samtools) were successfully compiled during the installation process, I had to modify the web links for the other tools (HISAT2, StringTie, featureCounts) to download the appropriate binaries since they are available for both Linux and Mac OS X platforms. The only tool that remained challenging was Trinity, which has been deprecated from Homebrew and needs to be installed by using

Docker. As I did not manage to associate the execution of Trinity on the Docker with Necklace, I had to run them separately and modify the source code of the file "de_novo_assembly.groovy" to link the Trinity output file. Therefore, I suggest that Necklace would be also implemented on Mac OS X platform, so that a wider range of users could benefit from it.

We now provide an installation script for MacOS which has been tested. The installation instructions on our wiki have also been modified.

Specific comments related to Necklace:

1) Linked with what I previously mentioned, since Trinity requires a lot of computer resources to run, I wonder whether an option should be provided to bypass Trinity execution. In this case, users with limited computation resources could run Trinity on external servers, such as Galaxy, and then provide the Trinity output file directly to Necklace.

We have now implemented this option.

2) I think that the list of input parameters that one can provide to Necklace is too limited. Currently, one can only adjust the number of threads, the maximum memory allocated to Trinity and the score/identity thresholds for Blat alignment to build the superTranscriptome. For instances, indicating the strand specificity to HISAT2, StingTie and Trinity, or restricting the fragment counting to pairs with both reads mapped (featureCounts -B), would be more appropriate in certain cases. Given that these tools accept numerous parameters, the simplest and easiest way may be to allow users to provide a string with all wanted parameters and arguments for a given tool, thus preventing them to modify the Necklace source code to adapt their analysis. Another alternative could be to include a file of parameters that advanced users can modify at their own appreciation.

This is a great suggestion and we now provide command line options to pass various strings to the tools used by Necklace. These are documented at https://github.com/Oshlack/necklace/wiki/Options

3) Currently, the genome and annotation(s) of only one related species can be provided to Necklace. Could the authors modify this so that users can provide information from several related species? As compared to mammals, yeasts and flies, there is no bird species with an accurate genome sequence and annotation. Therefore, I think that performing the comparison of newly annotated gene candidates to a single related species may be too restrictive.

We thank the reviewer for this suggestion. We believe this can be addressed by running Necklace iteratively to include information from multiple species. We have now updated our documentation to explain this application (see the new FAQ section of the wiki).

4) Although I completely understand the strategy employed by the authors during the transcript clustering, I not sure whether this is the most appropriate approach. On one hand, candidate genes generated from de novo assembly must match either a gene detected during the genome-guided superTranscriptome, or a gene present in the related species superTranscriptome. This may result in the loss of a significant proportion of gene candidates if the studied organism has an incomplete genome and the related species is poorly annotated or too distant to be efficiently compared at the nucleotide level. For example, it has been shown that genes encoding long non-coding RNAs have rapidly diverged through evolution as compared to protein-coding genes. On the other hand, the authors mentioned that de novo assembled transcripts mapped on more than one gene are also removed. Genomes that contain numerous gaps and unassigned contigs (e.g. chicken) tend to display a non-negligible proportion of fragmented genes, i.e. spanning on multiple contigs, of which each region is erroneously annotated as a distinct gene. Thus, de novo assembled transcripts spanning on several regions of a fragmented gene would be wrongly defined as false chimera and be subsequently ignored. Could the authors discuss these two points?

This is a valid concern and we accept that our approach may miss novel transcribed sequence and/or be unable to reconstruct "fragmented" genes, which are two of the strengths of de novo transcriptome assembly. However, very often the assembled contigs that are inconsistent or absent from both the reference genome and a related species, are assembly errors. For example, false chimeras are an extremely common assembly artefact that give the false appearance of a "fragmented" gene. In Necklace we have intentionally made the decision to prioritise correctness over completeness and we still manage to recover novel genes in all our example data sets. In addition we have added a discussion of this point in the text in the sections: "Clustering of transcripts" and "Conclusion".

Specific comments for the manuscript:

1) I suggest to the authors to further expand the current limitations of RNA-seq analysis on poorly annotated organisms in the introduction. This would help the non-specialist readers to clearly identify the usefulness of Necklace for their RNA-seq data analysis.

We have now expanded the introduction with this suggestion.

2) Figure 1: the arrowhead spanning from the "de novo assembly" box to the clustering is hidden. Could the authors also highlight differently tools and file formats? Both are in bold.

We have corrected this.

3) Page 4, line 18: "superTrascriptome" should be replaced by "superTranscriptome".

We have corrected this.

4) Could the authors describe the current version of the Churra sheep milk genome (Oar_v3.1) that they used for validation? In terms of genome size, number of gaps, number of sequenced chromosomes, number of unassigned contigs

We have added this information to the manuscript in the section "Application to differential expression testing in sheep transcriptomes".

5) Could the authors give more details on their results with the sheep transcriptome? Could they assign a function to the 2,208 additional genes identified and to the 66 differentially expressed genes newly detected? Are they known protein-coding genes? Do they encode putative proteins or non-coding RNAs? This would highlight the potential benefits of running Necklace on partially annotated genomes. I also suggest to the authors to include a table summarizing the results, in order to help the reader.

We now include these details and provide a summary table.

6) Page 5, lines 6 and 7 (as well as legend of figure 2): "321bp" and "3333bp" should be written "321 bp" and "3333 bp", respectively.

We have corrected this.

7) I think authors should expand further in their conclusions what are the strengths of Necklace. The idea of combining genome-guided and de novo assemblies prior to RNA-seq analysis is not new, but Necklace is the first automated pipeline to my knowledge that is user-friendly, allows a high reproducibility and seems adaptable to a larger scale. However, it is also noteworthy to mention the potential limitations encountered when analysing RNA-seq data on highly fragmented genomes or from organisms with no closely-related species with accurate gene annotation.

We have now expanded the Conclusions section with these suggestions.

8) In the methods section, could the authors indicate the time of execution for the different commands?

We now include this information.

List of abbreviations (page 8): "fragements" should be corrected to "fragments".

We have corrected this.
