# Supplementary material for: Necklace: combining reference and assembled transcriptomes for more comprehensive RNA-Seq analysis
Source: Gigascience. 2018 May 2;7(5):giy045. doi: 10.1093/gigascience/giy045 (PMC5946861; doi:10.1093/gigascience/giy045)
Supplement: GIGA-D-17-00354_R2.pdf [file giy045_giga-d-17-00354_r2.pdf]

GigaScience

Necklace: combining reference and assembled transcriptomes for more comprehensive RNA-Seq analysis

--Manuscript Draft--

**Manuscript Number:**	GIGA-D-17-00354R2
**Full Title:**	Necklace: combining reference and assembled transcriptomes for more comprehensive RNA-Seq analysis
**Article Type:**	Technical Note
**Funding Information:**	National Health and Medical Research Council (GNT1126157) — Prof. Alicia Oshlack
**Abstract:**	Background: RNA-Seq analyses can benefit from performing a genome-guided and de novo assembly, in particular for species where the reference genome or the annotation is incomplete. However, tools for integrating assembled transcriptome with reference annotation are lacking.<br>Findings: Necklace is a software pipeline that runs genome-guided and de novo assembly and combines the resulting transcriptomes with reference genome annotations. Necklace constructs a compact but comprehensive superTranscriptome out of the assembled and reference data. Reads are subsequently aligned and counted in preparation for differential expression testing.<br>Conclusions: Necklace allows a comprehensive transcriptome to be built from a combination of assembled and annotated transcripts which results in a more comprehensive transcriptome for the majority of organisms. In addition RNA-seq data is mapped back to this newly created superTranscript reference to enable differential expression testing with standard methods. Necklace is available from https://github.com/Oshlack/necklace/wiki under GPL 3.0.
**Corresponding Author:**	Alicia Oshlack<br><br>AUSTRALIA
**Corresponding Author Secondary Information:**	
**Corresponding Author's Institution:**	
**Corresponding Author's Secondary Institution:**	
**First Author:**	Nadia Davidson
**First Author Secondary Information:**	
**Order of Authors:**	Nadia Davidson
	Alicia Oshlack
**Order of Authors Secondary Information:**	
**Response to Reviewers:**	Reviewer #1: The authors have addressed my concerns.<br><br>Here are a few more minor comments.<br><br>(1) There are still several places of inconsistency of the capitalization of first letter for Necklace. For example, the first paragraph in the conclusion section uses "necklace", while in the second paragraph, the authors use "Necklace".<br><br>We have now corrected these inconsistencies.<br><br>(2) It seems Necklace will filter a de novo assembled transcript if it can not be found in reference-based superTranscriptome or related species. Can the authors emphasize this point in the first paragraph of "clustering of transcripts" to make the description of filtration complete.

Powered by Editorial Manager® and ProduXion Manager® from Aries Systems Corporation

	We have added a statement as suggested.<br><br>(3) For the 2208 novel genes identified in the sheep example, can the authors explained more about 1621 genes that can not be verified by blastp? Could their sequence be identified by blastn on refseq-rna database? If most of the genes are false positive, Necklace could add a step to filter the false genes by blast in future.<br><br>We thank the reviewer for this excellent suggestion. Blastn with the refseq-rna database showed that most of the unidentified genes can be annotated as potential coding genes. We have now included these results in the results and method section of our manuscript.<br><br><br>Reviewer #2: As previously stated, I believe that Necklace will be valuable for numerous users dealing with RNA-seq data generated from incomplete and poorly annotated genomes. I particularly appreciate the efforts made by the authors to provide a version of Necklace executable on Mac OS X platform, as well as the implementation of options to adjust the analysis. I have tested this new version on my own Mac OS X server (while passing a set of Trinity contigs generated independently) and it worked well. With the revised version of their manuscript, authors addressed my concerns and improved the clarity of their approach. I consider it is now acceptable for publication.<br><br>Minor comment:<br>When testing this new version of Necklace, I have figured out a feature that I missed during the initial review. If the filenames of input reads have a format such as "baseName_ID1_R1/2.fastq.gz, baseName_ID2_R1/2.gz", then only one alignment map is created "baseName.bam" during the read re-alignment against the superTranscriptome, which turns out to only contain the reads from the first input files "baseName_ID1_R1/2.fastq.gz". Consequently, read counting is only performed afterwards for these reads. It is not really clear from the Wiki page that Necklace accepts a specific format for the read filenames, where the unique sample name ID should be placed prior to any "_".<br><br>We thank the reviewer for identifying this issue. In fact, Necklace does include an option to specify the format of the read filenames, but we had neglected to document this. We now describe it on our wiki “Options” page and make the default format that is expected more explicit in our “Getting Started” wiki page.
**Additional Information:**	
**Question**	**Response**
Are you submitting this manuscript to a special series or article collection?	No
**Experimental design and statistics**<br><br><br>Full details of the experimental design and statistical methods used should be given in the Methods section, as detailed in our Minimum Standards Reporting Checklist. Information essential to interpreting the data presented should be made available in the figure legends.<br><br><br>Have you included all the information requested in your manuscript?	Yes
**Resources**	Yes

Powered by Editorial Manager® and ProduXion Manager® from Aries Systems Corporation

A description of all resources used, including antibodies, cell lines, animals and software tools, with enough information to allow them to be uniquely identified, should be included in the Methods section. Authors are strongly encouraged to cite Research Resource Identifiers (RRIDs) for antibodies, model organisms and tools, where possible.<br><br>Have you included the information requested as detailed in our Minimum Standards Reporting Checklist?	
**Availability of data and materials**<br><br>All datasets and code on which the conclusions of the paper rely must be either included in your submission or deposited in publicly available repositories (where available and ethically appropriate), referencing such data using a unique identifier in the references and in the "Availability of Data and Materials" section of your manuscript.<br><br>Have you have met the above requirement as detailed in our Minimum Standards Reporting Checklist?	Yes

Manuscript

Click here to download Manuscript Necklace Gigascience_r4.docx

Necklace: combining reference and assembled transcriptomes for more comprehensive RNA-Seq analysis

Nadia M Davidson[1,2,*] and Alicia Oshlack[1,2,*]
[1]Murdoch Childrens Research Institute, Royal Children's Hospital, Victoria, Australia
[2]School of Bio-Sciences, University of Melbourne, Victoria, Australia
*To whom correspondence should be addressed.
Contact: nadia.davidson@mcri.edu.au or alicia.oshlack@mcri.edu.au (ORCID: 0000-0001-9788-5690)

Abstract

Background: RNA-Seq analyses can benefit from performing a genome-guided and de novo assembly, in particular for species where the reference genome or the annotation is incomplete. However, tools for integrating assembled transcriptome with reference annotation are lacking.
Findings: Necklace is a software pipeline that runs genome-guided and de novo assembly and combines the resulting transcriptomes with reference genome annotations. Necklace constructs a compact but comprehensive superTranscriptome out of the assembled and reference data. Reads are subsequently aligned and counted in preparation for differential expression testing.
Conclusions: Necklace allows a comprehensive transcriptome to be built from a combination of assembled and annotated transcripts which results in a more comprehensive transcriptome for the majority of organisms. In addition RNA-seq data is mapped back to this newly created superTranscript reference to enable differential expression testing with standard methods. Necklace is available from https://github.com/Oshlack/necklace/wiki under GPL 3.0.

Keywords: transcriptome, assembly, RNA-Seq, non-model

Findings

Introduction

Despite the increasing number of species with a sequenced genome, the vast majority of reference genomes are incomplete. They may contain gaps, have unplaced assembly scaffolds and be poorly annotated. The naïve approach to analysing RNA-Seq on species with a genome would follow the same procedure as model organisms; align reads the genome and count reads overlapping annotated genes, then test for differential expression based on gene counts [1]. However, this approach has the potential to miss important biology for many organisms. Segments of genes may be missed, either because of a gap in the reference sequence or missing annotation. The downstream differential expression analysis is likely to have reduced statistical power because the gene counts are underestimated. Similarly, we have observed different segments of a gene being annotated as separate genes such as when the gene spans multiple

assembly scaffolds. However, this can happen even when a gene sits within a single scaffold. In the worst case, whole genes can be missed.

Ideally, an RNA-Seq analysis could repair the gene-models available from a reference genome and annotation, by extracting information about the expressed genes from the data itself through genome-guided and/or de novo assembly [2]. However, analyses involving assembly remain complex, more so when multiple assemblies need to be integrated. Prior works such as [3] have gone some way to addressing the challenge, however no reusable software has been written to perform these types of analyses.

In Davidson et al. 2017, [4] we introduced the concept of the superTranscriptome, where each gene is represented by one sequence containing all of that gene's exons in transcriptional order. SuperTranscripts provided a convenient means in which transcriptomes from difference sources, such as assembly and annotation, can be combined into a compact and unified reference. When applied to chicken, we showed that we could recover hundreds of segments of genes that were absent from the chicken reference genome.

Here we present software called Necklace which automates the process described in [4] for any species with an incomplete reference genome. Necklace takes as input a configuration file containing paths to the RNA-seq reads, a reference genome and one or more reference genome annotation. Because de novo assembly is error prone, we require that any gene discovered specifically through de novo assembly be also found amongst the coding sequence of a related (well annotated) species. Therefore, the genome and annotation of a related species must also be provided to Necklace. Necklace will then run the steps involved in genome-guided and de novo assembly, and combine the assembled transcriptome with reference annotations for the species of interest. After building the superTranscriptome, Necklace will align and count reads in preparation for testing for differential gene expression and differential transcript usage using well established tools such as edgeR [5], DEseq[6] or DEXseq[7].

In order to demonstrate the application of Necklace in a new data set we analysed public RNA-seq data from sheep milk. Compared to using the sheep reference genome on it own, the Necklace analysis resulted in 18% more reads being assigned to genes and 19% more differentially expressed genes being detected.

The Necklace pipeline

Necklace is a pipeline constructed using the bpipe framework [8]. It steers external software, such as aligners and assemblers, as well as a set of its own utilities, written in C/C++. As input Necklace takes the raw RNA-seq reads and the reference genome for the species as well as any available annotation. In addition, it takes a reference genome and annotation from a related, but well studied species such as human, drosophila, yeast, etc. Necklace consists of several sequential stages: initial genome guided and *de novo* assembly, clustering transcripts into gene groupings, reassembly to build the superTranscriptome

and finally alignment and counting of mapped reads in preparation for differential expression testing and differential isoform usage testing. Each of these sequential stages consists of several sub-stages and is outlined in Figure 1 with further detail below.

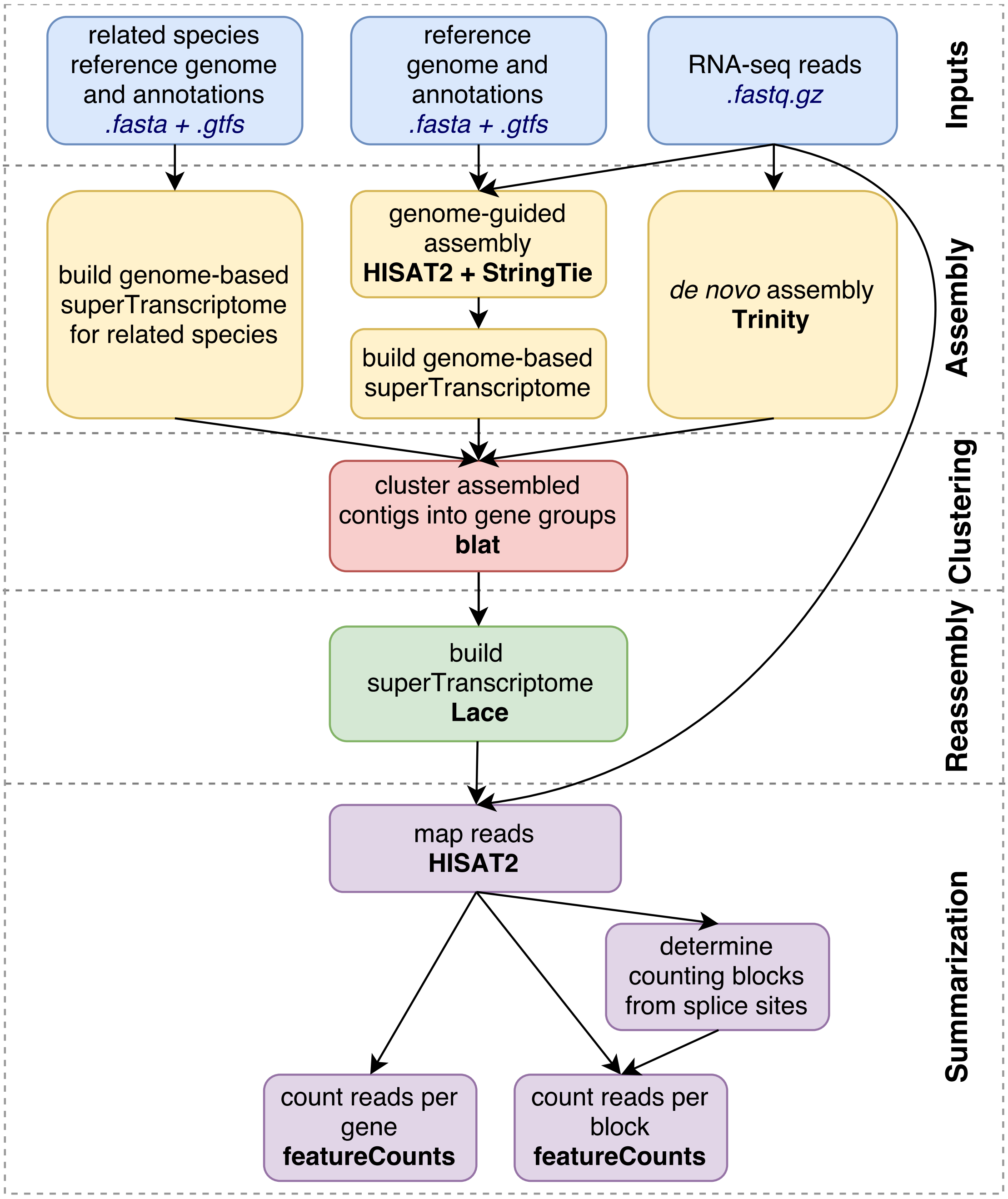

Figure 1. *An overview of the Necklace pipeline. External software that Necklace runs is shown in bold.*

Assembly

The assembly stage creates three different transcriptomes. First reads are aligned to the reference genome using HISAT2 [9] and genome-guided assembly is performed with StringTie [10]. This assembly is combined with reference annotations and then flattened based on genomic location, so that each exon is

reported only once and overlapping exons are merged. Exonic sequence is then extracted from the genome and concatenated to build a "genome-based" superTranscriptome. We build the initial genome-based superTranscriptome rather than use a set of transcript sequences for two reasons. Firstly, it results in the correct genomic order of a gene's exons. This ensures that when Lace is used in the reassembly step to combine the de novo assembled transcripts, the final superTranscriptome also has exons in the correct genomic order. Secondly, this step reduces the amount of sequence to be processed in the Clustering and Reassembly Steps.

In parallel to building the genome-based superTranscriptome for the species of interest, the related species annotation is used to create a genome-based superTranscriptome (without genome-guided assembly). Finally, RNA-Seq reads are de novo assembled with Trinity [11].

Clustering of transcripts

This step assigns de novo assembled transcripts to gene clusters prior to building the final superTranscriptome. Those contigs aligning to the genome-based superTranscriptome (using Blat [12]) are allocated to known genes while those not aligning to the genome, but found in the related species superTranscriptome are assigned to novel genes. De novo assembled contigs that are not found in either the genome-based superTranscriptome or a related species are filtered out. De novo assembled transcripts that align to more than one gene are also removed to avoid false chimeras [13] from being introduced into the superTranscriptome.

A limitation of this filtering is that de novo assembled contigs cannot be used to scaffold highly fragmented references because those contigs will give the appearance of false chimeras. Novel genes which are absent from the genome and the related species will also be missed with our approach. However, de novo assemblies are highly error prone and strict filtering of assembled transcripts is required to eliminate the introduction of artifacts into the annotation.

Reassembly of superTranscripts

Each cluster consists of a gene's genome-based superTranscript and/or its set of de novo assembled transcripts. The transcripts in each cluster are merged together through Lace assembly [4], to produce one superTranscript per gene.

Summarization

Reads are aligned back to the superTranscriptome using HISAT2 and fragments counted per gene using featureCounts [14]. Splice junctions reported by HISAT2 are used to segment each superTranscript into a set of contiguous "blocks". Fragments are then counted in "blocks" and can be used for differential isoform detection like exon counts.

Application to differential expression testing in sheep transcriptomes

To demonstrate the utility of Necklace, we applied it to public RNA-Seq from Churra sheep milk and compared transcriptome expression at day 10 to day 150

post lambing [15]. Necklace was given the sheep reference genome, Oar_v3.1. This version of the sheep genome is 2.6 GB in size, with 85 MB of unfilled assembly gaps. It consists of 5,698 scaffolds (28 chromosomes and 5,670 unplaced contigs). Human, with the hg38 reference genome, was used as the related species. For both genomes, version 90 of the Ensembl annotation was used (see methods).

The Ensembl reference consisted of 29,118 transcripts and reference guided assembly using StringTie resulted in 65,717 transcripts. The sheep data was *de novo* assembled into 267,553 contigs, however only 63,592 contigs were reassembled into the Necklace superTranscriptome due to filtering at the clustering step. The magnitude of this reduction is consistent with alternative clustering methods (e.g. the removal of contigs with little read support using Corset [13]).

Using this data and set of reference files resulted in a more comprehensive transcriptome. Compared to the Ensembl sheep annotation, the number of bases included in the Necklace transcriptome increased by 76% and 18% more reads were assigned to genes by featureCounts (Table 1). This more comprehensive reference included 2208 (8%) more genes. A small subset of the novel genes (404 genes) were assembled in the antisense direction or overlapping an annotated Ensembl gene. Of the remaining novel genes, 1718 (95%) had homology to sequence in the RefSeq RNA database [16] with an expect value (E) below 10^{-50} when aligned using blastn [17]. The novel genes predominately matched coding (86%) rather than non-coding (14%) sequence. For 381 novel genes an open reading frame of 100 or more amino acids was identified with homology to protein sequence from another species.

When performing differential expression analysis using edgeR the Necklace transcriptome identified more significantly differentially expressed genes than using the reference alone (456 compared to 383, FDR<0.05). Some of these differences could be attributed to the inclusion of novel unannotated genes, with 66 of the newly annotated genes identified as differentially expressed. A predicted protein coding gene could be assigned to 45 of the novel differentially expressed genes after blastn alignment to the RefSeq RNA database . Necklace was also able to improve the detection of differential expression amongst several known genes by providing more complete gene sequences. Larger numbers of reads mapping to the longer sequences resulted in more power for differential expression testing. One example of this was the *SERTM1* gene where the annotated transcript only included 321 bp while the Necklace superTranscript contained 3333 bp and overlapped a genome assembly gap (Figure 2).

	**Reference**	**Necklace**
**Bases (Mbp)**	45.13	79.56
**Reads assigned to genes**	194,693,051	230,140,801
**Genes**	26,613	28,821
**Differentially expressed genes**	383	456

Table 1. *A comparison of using the Ensembl reference annotation alone and using the superTranscriptome generated by Necklace for our example sheep dataset.*

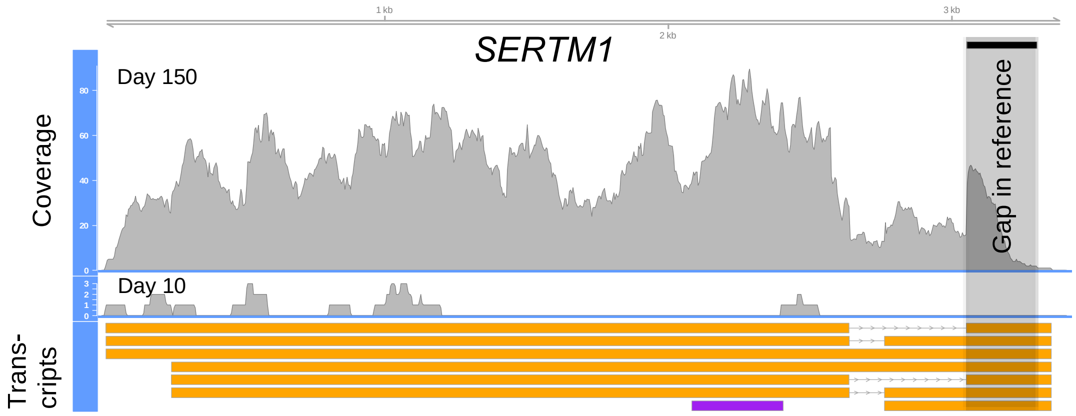

Figure 2. *Read coverage aggregated over replicate samples for the Necklace assembled superTranscript of SERTM1. This gene is found to be significantly differentially expressed using the Necklace generated reference, but is missed when the reference genome and annotation are used in isolation due to low read counts. The reference annotation consists of a single transcript of 321 bp (shown in purple), whereas the de novo assembled gene consists of seven transcripts up to 3333 bp long (shown in orange) and includes approximately 250bp that is absent from the reference genome, in a location consistent with a genome assembly gap. The genome-guided transcripts that were assembled for this gene were filtered out by StringTie's merge function due to an average FPKM < 1.*

Conclusion

Here we have presented Necklace, a pipeline designed to improve RNA-seq analysis in species with an incomplete genome and annotation. We believe Necklace is the first pipeline to automate the steps required to combine reference and assembled data, alignment and summarization of counts thereby making the analysis process user friendly and reproducible.

Incorporating *de novo* assembled data into the analysis of species with a semi-complete genome promises to give a more comprehensive picture of the transcriptome. However, de novo assemblies can also introduce artifacts and for this reason Necklace only adds de novo assembled transcripts corresponding to novel protein coding genes which are found in a related species. Assembled transcripts that match multiple known genes are also removed, in order to prevent the introduction of false chimeric sequences. Therefore it is still possible that some *bona fide* expressed transcripts are not included in the final results.

In real data, we show that indeed applying Necklace to a sheep data set resulted in a more complete transcriptome. We were able to discover more exon and more genes, some of which had homology to known protein coding sequences in other species. This analysis also resulted in more power for differential expression analysis. Necklace is open source and available from github at https://github.com/Oshlack/necklace/wiki and has resource RRID:SCR_016103.

Methods

Data

Sheep RNA-Seq data was downloaded from SRA (accession numbers SRR2932539- SRR2932542,SRR2932561-SRR2932564). The sheep genome and annotation was downloaded from Ensembl:
ftp://ftp.ensembl.org/pub/release-90/fasta/ovis_aries/dna/Ovis_aries.Oar_v3.1.dna.toplevel.fa.gz
ftp://ftp.ensembl.org/pub/release-90/gtf/ovis_aries/Ovis_aries.Oar_v3.1.90.gtf.gz

The human reference genome and annotation was also downloaded from Ensembl:
ftp://ftp.ensembl.org/pub/release-90/fasta/homo_sapiens/dna/Homo_sapiens.GRCh38.dna.toplevel.fa.gz,
ftp://ftp.ensembl.org/pub/release-90/gtf/homo_sapiens/Homo_sapiens.GRCh38.90.gtf.gz

We then selected coding sequence from the human annotation using the command:

```
grep " CDS " data/Homo_sapiens.GRCh38.90.gtf > Homo_sapiens.GRCh38.90.CDS.gtf
```

Necklace Analysis

For the Necklace analysis of sheep milk, all data files were placed into a subdirectory called "data" and a Necklace input file, "data.txt", was created with the following lines:

```
// sequencing data
reads_R1="data/SRR2932539_1.fastq.gz,data/SRR2932540_1.fastq.gz,data/SRR2932541_1.fastq.gz,data/SRR2932542_1.fastq.gz,data/SRR2932561_1.fastq.gz,data/SRR2932562_1.fastq.gz,data/SRR2932563_1.fastq.gz,data/SRR2932564_1.fastq.gz"
reads_R2="data/SRR2932539_2.fastq.gz,data/SRR2932540_2.fastq.gz,data/SRR2932541_2.fastq.gz,data/SRR2932542_2.fastq.gz,data/SRR2932561_2.fastq.gz,data/SRR2932562_2.fastq.gz,data/SRR2932563_2.fastq.gz,data/SRR2932564_2.fastq.gz"

//The genome and annotation
```

```
annotation="data/Ovis_aries.Oar_v3.1.90.gtf"
genome="data/Ovis_aries.Oar_v3.1.dna.toplevel.fa"

//The genome and annotation of a related species
annotation_related_species="data/Homo_sapiens.GRCh38.90.CDS.gtf"
genome_related_species="data/Homo_sapiens.GRCh38.dna.toplevel.fa"
```

Necklace version 0.9 was then run using the command:

```
<necklace path>/tools/bin/bpipe run -n 8 <necklace path>/necklace.groovy
data/data.txt
```

Version numbers of all the external tools that Necklace calls can be found in Necklace's installation script, "install_linux64.sh".

Necklace was run on 8 cores on a 48 core AMD Opteron(tm) Processor 6180 SE 2.5 GHz CentOS 6.7 server with 252GB of RAM. The full run time was approximately 4.5 days.

The assembly stage took approximately 3.5 days due to the *de novo* assembly (Trinity [11] was run with 6 threads and 50 GB memory maximum). Genome-guided assembly was run concurrently on 1 core and took 20 hours. The time for clustering was 11 hours, which was dominated by alignment of the *de novo* assembled contigs to the related species with blat. Lace ran in 13 hours and read realignment and summarization took 1.5 hours.

Annotation of Novel Genes

Novel superTranscript sequences were aligned to the RefSeq RNA database (downloaded from ftp://ftp.ncbi.nlm.nih.gov/blast/db/) using blastn version 2.2.25+ and the command,

```
blastn –db refseq_rna –outfmt 6 –query novel_ST.fasta -num_alignments 1
```

StringTie and Trinity sequence from novel sheep genes were extracted and analysed with TransDecode [18]and blastp (BLASTP, RRID:SCR_001010)[19] against the UniProt (UniProt , RRID:SCR_002380)[20] database using the commands:

```
TransDecoder.LongOrfs -t <transcripts.fasta>

blastp -query transdecoder_dir/longest_orfs.pep  \
   -db uniprot_sprot.fasta  -max_target_seqs 1 \
   -outfmt 6 -evalue 1e-5 -num_threads 10 > blastp.outfmt6

hmmscan --cpu 8 --domtblout pfam.domtblout /path/to/Pfam-A.hmm
transdecoder_dir/longest_orfs.pep

TransDecoder.Predict -t target_transcripts.fasta --retain_pfam_hits
pfam.domtblout --retain_blastp_hits blastp.outfmt6
```

Reference Based Analysis

To make the reference based analysis as similar as possible to the Necklace pipeline we used the versions of HISAT2 (HiSat2, RRID:SCR_015530), samtools (SAMTOOLS, RRID:SCR_002105) and featureCounts (featureCounts, RRID:SCR_012919) that were installed by Necklace.

HISAT2 was run on each sample using the command:

```
hisat2 --known-splicesite-infile <splice sites file> -x <genome index> -1 <input_1.fastq.gz> -2 <input_2.fastq.gz> | samtools view -u - > <output.bam>
```

Where the splice sites file and genome index were the same ones generated in the initial stage of Necklace that aligns reads to the reference genome.

Reads were then counted for each annotated gene using featureCounts with the command:

```
featureCounts -T 8 --primary -p -t exon -g gene_id -a Ovis_aries.Oar_v3.1.90.flat.gtf -o counts *.bam
```

Where "Ovis_aries.Oar_v3.1.90.flat.gtf" was a flattened version of the sheep Ensembl annotation and was created with the Necklace command:

```
gtf2flatgtf Ovis_aries.Oar_v3.1.90.gtf Ovis_aries.Oar_v3.1.90.flat.gtf
```

Flattening the annotation involves merging transcripts of a gene into a non-redundant but complete set of exons.

Differential Expression Testing

For differential gene expression testing, gene-level counts were analysed using the R bioconductor package edgeR (version 3.18.1) (edgeR, RRID:SCR_012802) [21]. We modeled both the time-point post lambing and animal in the design matrix:

```
time_point<-c(rep("Day10",4),rep("Day150",4))
indv<-c(3141,4860,49537,9539,3141,4860,9539,49537) //numbers are animal IDs
design <- model.matrix(~0+factor(indv)+factor(time_point))
colnames(design) <- gsub("factor","",colnames(design))
sample_names=paste(indv,time_point,sep="_")
rownames(design)=sample_names
```

The counts table was read into R and passed to edgeR:

```
counts=count_table[,7:ncol(count_table)]
y <- DGEList(counts=counts)
```

Genes with a counts per million (cpm) less than or equal to 0.5 in less 4 samples were filtered out and the libraries normalized.

```
keep <- rowSums(cpm(y) > 0.5) >=4
y <- y[keep, , keep.lib.sizes=TRUE]
```

```
y <- calcNormFactors(y)
```

We then estimated the dispersion and looked for differential expression with a false discovery rate (FDR) < 0.05:

```
y <- estimateDisp(y,design,robust=TRUE)
fit <- glmFit(y, design,robust=TRUE)
qlf <- glmLRT(fit,coef=5)
is.de <- decideTests(qlf, p.value=0.05)
```

Availability of supporting source code and requirements

Project name: Necklace

Scicrunch RRID: SCR_016103

Project home page: https://github.com/Oshlack/necklace/wiki

Operating system(s): Linux

Programming language: Groovy and C/C++

Other requirements: Java 1.8

License: GPL 3.0

Availability of supporting data

An archival snapshot of the code is available in the GigaScience GigaDB repository[22].

Declarations

List of abbreviations

cpm - counts per million; FDR – False Discovery Rate; FPKM – fragments per kilobase of exon per million mapped reads

Competing interests

None declared.

Funding

AO is funded by an *NHMRC CDF GNT1126157.*

Authors' contributions

ND wrote all the software and drafted the paper. AO oversaw the project and contributed to writing the manuscript.

Acknowledgements
We would like to thank Anthony Hawkins, the author of Lace, who contributed to the early concept of Necklace when applied to chicken.

References
1. Oshlack A, Robinson MD, Young MD. From RNA-seq reads to differential expression results. Genome Biol.. 2010 [cited 2013 Mar 2];11:220. Available from: http://genomebiology.com/2010/11/12/220
2. Martin J, Wang Z. Next-generation transcriptome assembly. Nat. Rev. Genet. 2011;12:671–82. doi:10.1038/nrg3068
3. Orgeur M, Martens M, Börno ST, Timmermann B, Duprez D, Stricker S. A dual transcript-discovery approach to improve the delimitation of gene features from RNA-seq data in the chicken model. Biol. Open 2017; bio.028498. Available from: http://www.ncbi.nlm.nih.gov/pubmed/29183907
4. Davidson NM, Hawkins ADK, Oshlack A. SuperTranscripts: a data driven reference for analysis and visualisation of transcriptomes. Genome Biol. 2017 181 201718:148. Available from: doi:10.1186/s13059-017-1284-1
5. Robinson M, McCarthy D, Chen Y, Smyth GK. edgeR: differential expression analysis of digital gene expression data User's Guide. 2011;
6. Anders S, Huber W. Differential expression analysis for sequence count data. Genome Biol. 2010;11:R106. Available from: http://genomebiology.com/2010/11/10/R106
7. Anders S, Reyes A, Huber W. Detecting differential usage of exons from RNA-seq data. Genome Res. 2012;22:2008–17. Available from: http://www.ncbi.nlm.nih.gov/pubmed/22722343
8. Sadedin SP, Pope B, Oshlack A. Bpipe: a tool for running and managing bioinformatics pipelines. Bioinformatics. 2012 [cited 2013 Nov 15];28:1525–6. Available from: http://bioinformatics.oxfordjournals.org/content/early/2012/04/11/bioinformatics.bts167.abstract
9. Kim D, Langmead B, Salzberg SL. HISAT: a fast spliced aligner with low memory requirements. Nat. Methods. 2015 [cited 2016 Sep 14];12:357–60. Available from: http://www.ncbi.nlm.nih.gov/pubmed/25751142
10. Pertea M, Pertea GM, Antonescu CM, Chang T-C, Mendell JT, Salzberg SL. StringTie enables improved reconstruction of a transcriptome from RNA-seq reads. Nat. Biotechnol. 2015;33:290. Available from: http://www.nature.com/nbt/journal/v33/n3/full/nbt.3122.html
11. Haas BJ, Papanicolaou A, Yassour M, Grabherr M, Blood PD, Bowden J, et al. De novo transcript sequence reconstruction from RNA-seq using the Trinity platform for reference generation and analysis. Nat. Protoc. 2013;8:1494–512. doi:10.1038/nprot.2013.084
12. Kent WJ. BLAT--the BLAST-like alignment tool. Genome Res. 2002 [cited 2013 May 29];12:656–64. Available from: http://www.pubmedcentral.nih.gov/articlerender.fcgi?artid=187518&tool=pmcentrez&rendertype=abstract
13. Davidson NM, Oshlack A. Corset: enabling differential gene expression analysis for de novo assembled transcriptomes. Genome Biol. 2014;15:410.

Available from: http://genomebiology.com/2014/15/7/410
14. Liao Y, Smyth GK, Shi W. featureCounts: an efficient general purpose program for assigning sequence reads to genomic features. Bioinformatics. 2014;30:923–30. Available from: http://www.ncbi.nlm.nih.gov/pubmed/24227677
15. Suárez-Vega A, Gutiérrez-Gil B, Klopp C, Tosser-Klopp G, Arranz J-J. Comprehensive RNA-Seq profiling to evaluate lactating sheep mammary gland transcriptome. Sci. Data 2016;3:160051. http://www.nature.com/articles/sdata201651
16. O'Leary NA, Wright MW, Brister JR, Ciufo S, Haddad D, McVeigh R, et al. Reference sequence (RefSeq) database at NCBI: current status, taxonomic expansion, and functional annotation. Nucleic Acids Res. 2016 [cited 2018 Apr 8];44:D733–45. Available from: http://www.ncbi.nlm.nih.gov/pubmed/26553804
17. Altschul S, Gish W, Miller W, Myers E, Lipman D. Basic local alignment search tool. J. Mol. Biol. 1990;215:403–10. doi:10.1016/S0022-2836(05)80360-2
18. Haas & Papanicolaou et al. TransDecoder (Find Coding Regions Within Transcripts) https://github.com/TransDecoder/TransDecoder/wiki
19. McGinnis S, Madden T. BLAST: at the core of a powerful and diverse set of sequence analysis tools. Nucleic Acids Res. 2004;32:W20–5. doi:10.1093/nar/gkh435
20. Bateman A, Martin MJ, O'Donovan C, Magrane M, Alpi E, Antunes R, et al. UniProt: the universal protein knowledgebase. Nucleic Acids Res. 2017;45:D158–69. doi:10.1093/nar/gkw1099
21. Robinson M, McCarthy D, Smyth G. edgeR: a Bioconductor package for differential expression analysis of digital gene expression data. Bioinformatics. 2010;26:139–40. doi:10.1093/bioinformatics/btp616
22. Davidson, N, M; Oshlack, A (2018): Supporting data for "Necklace: combining reference and assembled transcriptomes for more comprehensive RNA-Seq analysis" GigaScience Database. http://dx.doi.org/10.5524/100438
