# Supplementary material for: Necklace: combining reference and assembled transcriptomes for more comprehensive RNA-Seq analysis
Source: Gigascience. 2018 May 2;7(5):giy045. doi: 10.1093/gigascience/giy045 (PMC5946861; doi:10.1093/gigascience/giy045)
Supplement: Response_to_Reviewer_Comments_Original_Submission.pdf [file giy045_response_to_reviewer_comments_original_submission.pdf]

Author's Response To Reviewer Comments

Close

Editor comments:

In addition, please register any new software application in the SciCrunch.org database to receive a RRID (Research Resource Identification Initiative ID) number, and include this in your manuscript. This will facilitate tracking, reproducibility and re-use of your tool.

We have now registered Necklace with SciCrunch, RRID SCR_016103 and quote this ID in the conclusion of the manuscript.

Reviewer #1:

1. What's the purpose of the second stage "Clustering of transcripts"? Since the program Lace also has a clustering step with "Corset", the "Clustering of transcripts" stage seems redundant. Though the authors mentioned two benefits (add annotation information and filter wrong transcripts) of this stage, these two benefits can be achieved in some post-processing step.

Corset and Lace are separate programs, and only Lace is used in Necklace. Although Corset could be used for clustering prior to running Lace, we instead used a custom script that parses blat results and is tailored to accurately cluster cross-species transcripts.

The authors says Necklace assigns the de novo assembled transcript to the gene cluster, but in Figure 1 there is an arrow from "genome-based superTranscriptome" to this stage. I'm confused by the terms here. Does the "de novo assembled transcripts" here means only Trinity's output or the results from both StringTie and Trinity?

In the manuscript we refer to de novo assembled transcripts as being the contigs produced from Trinity assembly and genome guided assembly being transcripts assembled from StringTie. The clustering step involves assigning each de novo assembled transcript to a "genome-based superTranscript" from either the species being studied, or the related species. This step requires as input the genome-based superTranscriptome as well as the de novo assembly (as we align the de novo assembly against these). We represent this input relationship with the arrows in Figure 1. Genome-guided transcripts from StringTie are assigned to genes based on their position in the genome prior to building the genome-based superTranscriptome during the “Assembly” stage. In the “Reassembly” stage we only require the output of the clustering stage so we have updated the figure to remove the other arrows as suggested.

2. In the application, the authors gave the example of the improved of a gene where the reference genome is incomplete. I'm wondering how Necklace compares against this naive workflow:

(1) For each transcript from StringTie's output, extract its sequence (concatenate the sequences of its exons).

(2) Put the sequences from StringTie and sequences from Trinity together, and run Lace on this bigger

set of sequences.

In other words, this naive workflow is just a simple application of Lace. And this is similar to the section "Combining reference and de novo assembled transcriptome" from superTranscript's paper except that there is no annotation here. The comparison of Necklace and this naive workflow would be much fairer.

To clarify, Necklace uses the same workflow (with some minor improvements) as the one presented in the "Combining reference and de novo assembled transcriptome" section of our superTranscript paper. The main goal of this manuscript is to present the software which automates that workflow in a user friendly way. Executing the workflow described by the reviewer could potentially be challenging for a non-expert and indeed automating these steps is likely to give a pipeline very similar to Necklace. The main differences between the review's purposed pipeline and Necklace are:
- Necklace also uses reference annotation. Reference annotation will improve the gene-models, it allows novel transcripts to be annotated and its inclusion is a minor extension to the pipeline.
- Necklace builds a genome-based superTranscriptome. This step should not alter the completeness or correctness of the annotation, but is a convenience: 1) it reduces the amount of sequence that needs to be processed in the clustering and Lace steps, and 2) It ensures that Lace will output exons in genomic order. We have now added a statement to clarify this in the manuscript subsection "Assembly".
- Necklace uses purpose built clustering rather than Corset and only accepts novel genes which match a protein coding gene in the related species. This point also relates to review #2's comment 4. We now also address this point it in the manuscript in the sections: "Clustering of transcripts", "Application to…" and "Conclusion.

1. The pipeline downloads all the required software packages regardless of whether they exist in the system. As a result, the pipeline alone takes about 3G disk space. Can the user specify the path to already-installed software so that Necklace could directly use it?

Yes, it is possible to specify already installed software in Necklace's file, "tools.groovy". We have now made this clearer in our Installation documentation on our github wiki.

2. Is there a way to set some environment variable for Necklace? For example, on my server the default Java version is v1.7 while I can direct Java to other versions such as v1.8. The Trinity requires Java version at least 1.8, and even though I specify Java to v1.8 on my server but bpipe
still use the default one. I got around this problem by change Trinity's script to access the desired Java version. Can this be resolved in an easier fashion?

Because Trinity requires an environment with java version 1.8, Necklace also requires this version of java. A work around is now available as de novo assembly can be run outside of Necklace, for example with a different assembler that does not require java 1.8. as requested below. We also provide this suggestion on our new wiki section, "FAQ".

3. The software for alignment and transcriptome assembly is rapidly evolving. Will Necklace allow the users to use their own choice of software? For example, they can use Shannon over Trinity.

This is an excellent point. Because tools don't tend to use the same arguments, and input and output formats, it would be difficult to make Necklace work with any generic assembler in a user friendly way.

However, we acknowledge that users may want to use alternative software for their assemblies for a variety of reasons. Therefore we have now included an option whereby an assembly performed outside of Necklace (either de novo or genome-guided) can be passed to the pipeline and the default assembly is bypassed.

Minor comments:

On page 4 line 31 and on page 5, line 52, the first letter of "Necklace" should be "n" to be consistent, though I think the first letter of the software should always be capitalized.

On page 5, line 52, the "c/c++" should be capitalized to "C/C++".

On page 7, line 40, "stringTie" should be "StringTie".

We have now corrected these errors.

Reviewer #2:

Considering the vast majority of sequenced organisms with an incomplete and poorly annotated genome, I do think that Necklace would be of particular interest for a wide range of users. However, my main concern is related to the availability of Necklace, which is only developed for Linux. Being a Mac OS X user, I had to modify the source code of Necklace to install it on my own device. While the tools provided with their source code (cluster, make_blocks, samtools) were successfully compiled during the installation process, I had to modify the web links for the other tools (HISAT2, StringTie, featureCounts) to download the appropriate binaries since they are available for both Linux and Mac OS X platforms. The only tool that remained challenging was Trinity, which has been deprecated from Homebrew and needs to be installed by using Docker. As I did not manage to associate the execution of Trinity on the Docker with Necklace, I had to run them separately and modify the source code of the file "de_novo_assembly.groovy" to link the Trinity output file. Therefore, I suggest that Necklace would be also implemented on Mac OS X platform, so that a wider range of users could benefit from it.

We now provide an installation script for MacOS which has been tested. The installation instructions on our wiki have also been modified.

Specific comments related to Necklace:

1) Linked with what I previously mentioned, since Trinity requires a lot of computer resources to run, I wonder whether an option should be provided to bypass Trinity execution. In this case, users with limited computation resources could run Trinity on external servers, such as Galaxy, and then provide the Trinity output file directly to Necklace.

We have now implemented this option.

2) I think that the list of input parameters that one can provide to Necklace is too limited. Currently, one can only adjust the number of threads, the maximum memory allocated to Trinity and the score/identity thresholds for Blat alignment to build the superTranscriptome. For instances, indicating the strand

specificity to HISAT2, StingTie and Trinity, or restricting the fragment counting to pairs with both reads mapped (featureCounts -B), would be more appropriate in certain cases. Given that these tools accept numerous parameters, the simplest and easiest way may be to allow users to provide a string with all wanted parameters and arguments for a given tool, thus preventing them to modify the Necklace source code to adapt their analysis. Another alternative could be to include a file of parameters that advanced users can modify at their own appreciation.

This is a great suggestion and we now provide command line options to pass various strings to the tools used by Necklace. These are documented at https://github.com/Oshlack/necklace/wiki/Options

3) Currently, the genome and annotation(s) of only one related species can be provided to Necklace. Could the authors modify this so that users can provide information from several related species? As compared to mammals, yeasts and flies, there is no bird species with an accurate genome sequence and annotation. Therefore, I think that performing the comparison of newly annotated gene candidates to a single related species may be too restrictive.

We thank the reviewer for this suggestion. We believe this can be addressed by running Necklace iteratively to include information from multiple species. We have now updated our documentation to explain this application (see the new FAQ section of the wiki).

4) Although I completely understand the strategy employed by the authors during the transcript clustering, I not sure whether this is the most appropriate approach. On one hand, candidate genes generated from de novo assembly must match either a gene detected during the genome-guided superTranscriptome, or a gene present in the related species superTranscriptome. This may result in the loss of a significant proportion of gene candidates if the studied organism has an incomplete genome and the related species is poorly annotated or too distant to be efficiently compared at the nucleotide level. For example, it has been shown that genes encoding long non-coding RNAs have rapidly diverged through evolution as compared to protein-coding genes. On the other hand, the authors mentioned that de novo assembled transcripts mapped on more than one gene are also removed. Genomes that contain numerous gaps and unassigned contigs (e.g. chicken) tend to display a non-negligible proportion of fragmented genes, i.e. spanning on multiple contigs, of which each region is erroneously annotated as a distinct gene. Thus, de novo assembled transcripts spanning on several regions of a fragmented gene would be wrongly defined as false chimera and be subsequently ignored. Could the authors discuss these two points?

This is a valid concern and we accept that our approach may miss novel transcribed sequence and/or be unable to reconstruct “fragmented” genes, which are two of the strengths of de novo transcriptome assembly. However, very often the assembled contigs that are inconsistent or absent from both the reference genome and a related species, are assembly errors. For example, false chimeras are an extremely common assembly artefact that give the false appearance of a “fragmented” gene. In Necklace we have intentionally made the decision to prioritise correctness over completeness and we still manage to recover novel genes in all our example data sets. In addition we have added a discussion of this point in the text in the sections: “Clustering of transcripts” and “Conclusion”.

Specific comments for the manuscript:

1) I suggest to the authors to further expand the current limitations of RNA-seq analysis on poorly

annotated organisms in the introduction. This would help the non-specialist readers to clearly identify the usefulness of Necklace for their RNA-seq data analysis.

We have now expanded the introduction with this suggestion.

2) Figure 1: the arrowhead spanning from the "de novo assembly" box to the clustering is hidden. Could the authors also highlight differently tools and file formats? Both are in bold.

We have corrected this.

3) Page 4, line 18: "superTrascriptome" should be replaced by "superTranscriptome".

We have corrected this.

4) Could the authors describe the current version of the Churra sheep milk genome (Oar_v3.1) that they used for validation? In terms of genome size, number of gaps, number of sequenced chromosomes, number of unassigned contigs

We have added this information to the manuscript in the section “Application to differential expression testing in sheep transcriptomes”.

5) Could the authors give more details on their results with the sheep transcriptome? Could they assign a function to the 2,208 additional genes identified and to the 66 differentially expressed genes newly detected? Are they known protein-coding genes? Do they encode putative proteins or non-coding RNAs? This would highlight the potential benefits of running Necklace on partially annotated genomes. I also suggest to the authors to include a table summarizing the results, in order to help the reader.

We now include these details and provide a summary table.

6) Page 5, lines 6 and 7 (as well as legend of figure 2): "321bp" and "3333bp" should be written "321 bp" and "3333 bp", respectively.

We have corrected this.

7) I think authors should expand further in their conclusions what are the strengths of Necklace. The idea of combining genome-guided and de novo assemblies prior to RNA-seq analysis is not new, but Necklace is the first automated pipeline to my knowledge that is user-friendly, allows a high reproducibility and seems adaptable to a larger scale. However, it is also noteworthy to mention the potential limitations encountered when analysing RNA-seq data on highly fragmented genomes or from organisms with no closely-related species with accurate gene annotation.

We have now expanded the Conclusions section with these suggestions.

8) In the methods section, could the authors indicate the time of execution for the different commands?

We now include this information.

List of abbreviations (page 8): "fragements" should be corrected to "fragments".

We have corrected this.

Close
