# Supplementary material for: Necklace: combining reference and assembled transcriptomes for more comprehensive RNA-Seq analysis
Source: Gigascience. 2018 May 2;7(5):giy045. doi: 10.1093/gigascience/giy045 (PMC5946861; doi:10.1093/gigascience/giy045)
Supplement: Response_to_Reviewer_Comments_Revision_1.pdf [file giy045_response_to_reviewer_comments_revision_1.pdf]

Author's Response To Reviewer Comments

Close

Reviewer #1: The authors have addressed my concerns.

Here are a few more minor comments.

(1) There are still several places of inconsistency of the capitalization of first letter for Necklace. For example, the first paragraph in the conclusion section uses "necklace", while in the second paragraph, the authors use "Necklace".

We have now corrected these inconsistencies.

(2) It seems Necklace will filter a de novo assembled transcript if it can not be found in reference-based superTranscriptome or related species. Can the authors emphasize this point in the first paragraph of "clustering of transcripts" to make the description of filtration complete.

We have added a statement as suggested.

(3) For the 2208 novel genes identified in the sheep example, can the authors explained more about 1621 genes that can not be verified by blastp? Could their sequence be identified by blastn on refseq-rna database? If most of the genes are false positive, Necklace could add a step to filter the false genes by blast in future.

We thank the reviewer for this excellent suggestion. Blastn with the refseq-rna database showed that most of the unidentified genes can be annotated as potential coding genes. We have now included these results in the results and method section of our manuscript.

Reviewer #2: As previously stated, I believe that Necklace will be valuable for numerous users dealing with RNA-seq data generated from incomplete and poorly annotated genomes. I particularly appreciate the efforts made by the authors to provide a version of Necklace executable on Mac OS X platform, as well as the implementation of options to adjust the analysis. I have tested this new version on my own Mac OS X server (while passing a set of Trinity contigs generated independently) and it worked well. With the revised version of their manuscript, authors addressed my concerns and improved the clarity of their approach. I consider it is now acceptable for publication.

Minor comment:
When testing this new version of Necklace, I have figured out a feature that I missed during the initial review. If the filenames of input reads have a format such as "baseName_ID1_R1/2.fastq.gz, baseName_ID2_R1/2.gz", then only one alignment map is created "baseName.bam" during the read re-alignment against the superTranscriptome, which turns out to only contain the reads from the first input files "baseName_ID1_R1/2.fastq.gz". Consequently, read counting is only performed afterwards for these reads. It is not really clear from the Wiki page that Necklace accepts a specific format for the read filenames, where the unique sample name ID should be placed prior to any "_".

We thank the reviewer for identifying this issue. In fact, Necklace does include an option to specify the format of the read filenames, but we had neglected to document this. We now describe it on our wiki "Options" page and make the default format that is expected more explicit in our "Getting Started" wiki page.

Close
