# Supplementary material for: Necklace: combining reference and assembled transcriptomes for more comprehensive RNA-Seq analysis
Source: Gigascience. 2018 May 2;7(5):giy045. doi: 10.1093/gigascience/giy045 (PMC5946861; doi:10.1093/gigascience/giy045)
Supplement: Reviewer_1_Report_(Original_Submission) -- Li Song — 1/12/2018 Reviewed [file giy045_reviewer_1_report_(original_submission).pdf]

Reviewer Report

Title: Necklace: combining reference and assembled transcriptomes for more comprehensive RNA-Seq analysis

Version: Original Submission **Date:** 1/12/2018

Reviewer name: Li Song

Reviewer Comments to Author:

In this manuscript, the authors described a pipeline that improved the gene annotation from RNA-seq reads by combining the results of the reference-based transcriptome assembly and de novo transcriptome assembly. The improved gene annotation indeed helped the down-streaming analysis.

This pipeline looks solid and the tools in it are well studied in other publications. I still have two major concerns.

1. What's the purpose of the second stage "Clustering of transcripts"? Since the program Lace also has a clustering step with "Corset", the "Clustering of transcripts" stage seems redundant. Though the authors mentioned two benefits (add annotation information and filter wrong transcripts) of this stage, these two benefits can be achieved in some post-processing step.
The authors says Necklace assigns the de novo assembled transcript to the gene cluster, but in Figure 1 there is an arrow from "genome-based superTranscriptome" to this stage. I'm confused by the terms here. Does the "de novo assembled transcripts" here means only Trinity's output or the results from both StringTie and Trinity?

2. In the application, the authors gave the example of the improved of a gene where the reference genome is incomplete. I'm wondering how Necklace compares against this naive workflow:
(1) For each transcript from StringTie's output, extract its sequence (concatenate the sequences of its exons).
(2) Put the sequences from StringTie and sequences from Trinity together, and run Lace on this bigger set of sequences.
In other words, this naive workflow is just an simple application of Lace. And this is similar to the section "Combining reference and de novo assembled transcriptome" from superTranscript's paper except that there is no annotation here. The comparison of Necklace and this naive workflow would be much fairer.

The running of the pipeline was smooth and I got the expected results. But I'm wondering whether the following points could be clarified.

1. The pipeline downloads all the required software packages regardless of whether they exist in the system. As a result, the pipeline alone takes about 3G disk space. Can the user specify the path to already-installed software so that Necklace could directly use it?

2. Is there a way to set some environment variable for Necklace? For example, on my server the default Java version is v1.7 while I can direct Java to other versions such as v1.8. The Trinity requires Java version at least 1.8, and even though I specify Java to v1.8 on my server but bpipe still use the default one. I got around this problem by change Trinity's script to access the desired Java version. Can this be resolved in an easier fashion?

3. The software for alignment and transcriptome assembly is rapidly evolving. Will Necklace allow the users to use their own choice of software? For example, they can use Shannon over Trinity.

Minor comments:
On page 4 line 31 and on page 5, line 52, the first letter of "Necklace" should be "n" to be consistent, though I think the first letter of the software should always be capitalized.
On page 5, line 52, the "c/c++" should be capitalized to "C/C++".
On page 7, line 40, "stringTie" should be "StringTie".

Level of Interest

Please indicate how interesting you found the manuscript: An article of importance in its field

Quality of Written English

Please indicate the quality of language in the manuscript: Acceptable

Declaration of Competing Interests

Please complete a declaration of competing interests, considering the following questions:

- Have you in the past five years received reimbursements, fees, funding, or salary from an organisation that may in any way gain or lose financially from the publication of this manuscript, either now or in the future?
- Do you hold any stocks or shares in an organisation that may in any way gain or lose financially from the publication of this manuscript, either now or in the future?
- Do you hold or are you currently applying for any patents relating to the content of the manuscript?
- Have you received reimbursements, fees, funding, or salary from an organization that holds or has applied for patents relating to the content of the manuscript?
- Do you have any other financial competing interests?
- Do you have any non-financial competing interests in relation to this paper?

If you can answer no to all of the above, write 'I declare that I have no competing interests' below. If your reply is yes to any, please give details below.

I declare that I have no competing interests

I agree to the open peer review policy of the journal. I understand that my name will be included on my report to the authors and, if the manuscript is accepted for publication, my named report including any attachments I upload will be posted on the website along with the authors' responses. I agree for my report to be made available under an Open Access Creative Commons CC-BY license (http://creativecommons.org/licenses/by/4.0/). I understand that any comments which I do not wish to

be included in my named report can be included as confidential comments to the editors, which will not be published.

I agree to the open peer review policy of the journal

To further support our reviewers, we have joined with Publons, where you can gain additional credit to further highlight your hard work (see: https://publons.com/journal/530/gigascience). On publication of this paper, your review will be automatically added to Publons, you can then choose whether or not to claim your Publons credit. I understand this statement.

Yes
