# Supplementary material for: Necklace: combining reference and assembled transcriptomes for more comprehensive RNA-Seq analysis
Source: Gigascience. 2018 May 2;7(5):giy045. doi: 10.1093/gigascience/giy045 (PMC5946861; doi:10.1093/gigascience/giy045)
Supplement: Reviewer_2_Report_(Original_Submission) -- Mickael Orgeur — 1/16/2018 Reviewed [file giy045_reviewer_2_report_(original_submission).pdf]

Reviewer Report

Title: Necklace: combining reference and assembled transcriptomes for more comprehensive RNA-Seq analysis

Version: Original Submission **Date:** 1/16/2018

Reviewer name: Mickael Orgeur

Reviewer Comments to Author:

With their manuscript entitled "Necklace: combining reference and assembled transcriptomes for more comprehensive RNA-Seq analysis", Davidson and Oshlack address a major issue related to the analysis of RNA-seq data coming from inaccurately annotated organisms. It is indeed clear now that performing an upstream transcript discovery, rather than using the available reference genome and annotation, improves the accuracy of gene expression profiles, especially when dealing with incomplete and/or poorly annotated genomes. Necklace is an analysis pipeline that is: (1) very well structured, combining widely used and published tools with in-house tools; (2) nicely documented (installation and usage guide, short tutorial with demo data, description of output files); (3) freely available with its source code via the GitHub platform; and (4) easy to use with a single command that automatizes numerous steps necessary for RNA-seq data analysis. I have tested Necklace with my own RNA-seq data obtained from chicken samples and it seems to recover well full-length transcripts.Considering the vast majority of sequenced organisms with an incomplete and poorly annotated genome, I do think that Necklace would be of particular interest for a wide range of users. However, my main concern is related to the availability of Necklace, which is only developed for Linux. Being a Mac OS X user, I had to modify the source code of Necklace to install it on my own device. While the tools provided with their source code (cluster, make_blocks, samtools…) were successfully compiled during the installation process, I had to modify the web links for the other tools (HISAT2, StringTie, featureCounts…) to download the appropriate binaries since they are available for both Linux and Mac OS X platforms. The only tool that remained challenging was Trinity, which has been deprecated from Homebrew and needs to be installed by using Docker. As I did not manage to associate the execution of Trinity on the Docker with Necklace, I had to run them separately and modify the source code of the file "de_novo_assembly.groovy" to link the Trinity output file. Therefore, I suggest that Necklace would be also implemented on Mac OS X platform, so that a wider range of users could benefit from it.Specific comments related to Necklace:1) Linked with what I previously mentioned, since Trinity requires a lot of computer resources to run, I wonder whether an option should be provided to bypass Trinity execution. In this case, users with limited computation resources could run Trinity on external servers, such as Galaxy, and then provide the Trinity output file directly to Necklace.2) I think that the list of input parameters that one can provide to Necklace is too limited. Currently, one can only adjust the number of threads, the maximum memory allocated to Trinity and the score/identity thresholds for Blat alignment to build the superTranscriptome. For instances, indicating the strand specificity to HISAT2, StingTie and Trinity, or restricting the fragment counting to pairs with both reads mapped (featureCounts -B), would be more appropriate in certain cases. Given that these tools accept numerous parameters, the simplest and easiest way may be to allow users to provide a string with all wanted parameters and arguments for a given tool, thus preventing them to modify the Necklace source code to adapt their analysis. Another alternative could be to include a file of parameters that advanced users can modify at their own appreciation.3) Currently, the genome and annotation(s) of only one related species can be provided to Necklace. Could the authors modify this so that users can provide information from several related species? As compared to mammals, yeasts and flies, there is no bird species with an accurate genome sequence and annotation. Therefore, I think that performing the comparison of newly annotated gene candidates to a single related species may be too restrictive.4) Although I completely understand the strategy employed by the authors during the transcript clustering, I not sure whether this is the most appropriate approach. On one hand, candidate genes generated from de novo assembly must match either a gene detected during the genome-guided superTranscriptome, or a gene present in the related species superTranscriptome. This may result in the

loss of a significant proportion of gene candidates if the studied organism has an incomplete genome and the related species is poorly annotated or too distant to be efficiently compared at the nucleotide level. For example, it has been shown that genes encoding long non-coding RNAs have rapidly diverged through evolution as compared to protein-coding genes. On the other hand, the authors mentioned that de novo assembled transcripts mapped on more than one gene are also removed. Genomes that contain numerous gaps and unassigned contigs (e.g. chicken) tend to display a non-negligible proportion of fragmented genes, i.e. spanning on multiple contigs, of which each region is erroneously annotated as a distinct gene. Thus, de novo assembled transcripts spanning on several regions of a fragmented gene would be wrongly defined as false chimera and be subsequently ignored. Could the authors discuss these two points?Specific comments for the manuscript:1) I suggest to the authors to further expand the current limitations of RNA-seq analysis on poorly annotated organisms in the introduction. This would help the non-specialist readers to clearly identify the usefulness of Necklace for their RNA-seq data analysis.2) Figure 1: the arrowhead spanning from the "de novo assembly" box to the clustering is hidden. Could the authors also highlight differently tools and file formats? Both are in bold.3) Page 4, line 18: "superTrascriptome" should be replaced by "superTranscriptome".4) Could the authors describe the current version of the Churra sheep milk genome (Oar_v3.1) that they used for validation? In terms of genome size, number of gaps, number of sequenced chromosomes, number of unassigned contigs...5) Could the authors give more details on their results with the sheep transcriptome? Could they assign a function to the 2,208 additional genes identified and to the 66 differentially expressed genes newly detected? Are they known protein-coding genes? Do they encode putative proteins or non-coding RNAs? This would highlight the potential benefits of running Necklace on partially annotated genomes. I also suggest to the authors to include a table summarizing the results, in order to help the reader.6) Page 5, lines 6 and 7 (as well as legend of figure 2): "321bp" and "3333bp" should be written "321 bp" and "3333 bp", respectively.7) I think authors should expand further in their conclusions what are the strengths of Necklace. The idea of combining genome-guided and de novo assemblies prior to RNA-seq analysis is not new, but Necklace is the first automated pipeline to my knowledge that is user-friendly, allows a high reproducibility and seems adaptable to a larger scale. However, it is also noteworthy to mention the potential limitations encountered when analysing RNA-seq data on highly fragmented genomes or from organisms with no closely-related species with accurate gene annotation.8) In the methods section, could the authors indicate the time of execution for the different commands?List of abbreviations (page 8): "fragements" should be corrected to "fragments".

Level of Interest

Please indicate how interesting you found the manuscript: An article of importance in its field

Quality of Written English

Please indicate the quality of language in the manuscript: Needs some language corrections before being published

Declaration of Competing Interests

Please complete a declaration of competing interests, considering the following questions:

- Have you in the past five years received reimbursements, fees, funding, or salary from an organisation that may in any way gain or lose financially from the publication of this manuscript, either now or in the future?
- Do you hold any stocks or shares in an organisation that may in any way gain or lose financially from the publication of this manuscript, either now or in the future?

- Do you hold or are you currently applying for any patents relating to the content of the manuscript?
- Have you received reimbursements, fees, funding, or salary from an organization that holds or has applied for patents relating to the content of the manuscript?
- Do you have any other financial competing interests?
- Do you have any non-financial competing interests in relation to this paper?

If you can answer no to all of the above, write 'I declare that I have no competing interests' below. If your reply is yes to any, please give details below.

I declare that I have no competing interests.

I agree to the open peer review policy of the journal. I understand that my name will be included on my report to the authors and, if the manuscript is accepted for publication, my named report including any attachments I upload will be posted on the website along with the authors' responses. I agree for my report to be made available under an Open Access Creative Commons CC-BY license (http://creativecommons.org/licenses/by/4.0/). I understand that any comments which I do not wish to be included in my named report can be included as confidential comments to the editors, which will not be published.

I agree to the open peer review policy of the journal

To further support our reviewers, we have joined with Publons, where you can gain additional credit to further highlight your hard work (see: https://publons.com/journal/530/gigascience). On publication of this paper, your review will be automatically added to Publons, you can then choose whether or not to claim your Publons credit. I understand this statement.

Yes
